# An intrinsic purine metabolite AICAR blocks lung tumour growth by targeting oncoprotein mucin 1

**DOI:** 10.1038/s41416-023-02196-z

**Published:** 2023-02-21

**Authors:** Fareesa Aftab, Alice Rodriguez-Fuguet, Luis Silva, Ikei S. Kobayashi, Jiao Sun, Katerina Politi, Elena Levantini, Wei Zhang, Susumu S. Kobayashi, Wen Cai Zhang

**Affiliations:** 1 Department of Cancer Division, Burnett School of Biomedical Sciences, College of Medicine, University of Central Florida, 6900 Lake Nona Boulevard, Orlando, FL 32827 USA; 2 Department of Medicine, Beth Israel Deaconess Medical Center and Harvard Medical School, 330 Brookline Avenue, E/CLS-409, Boston, MA 02215 USA; 3 Department of Computer Science, College of Engineering and Computer Science, University of Central Florida, 4000 Central Florida Boulevard, Orlando, FL 32816 USA; 4 Departments of Pathology and Internal Medicine (Section of Medical Oncology) and the Yale Cancer Center, Yale University School of Medicine, New Haven, CT 06520 USA; 5 Harvard Stem Cell Institute, 330 Brookline Avenue, Harvard Medical School, Boston, MA 02215 USA; 6 Institute of Biomedical Technologies, National Research Council (CNR), Area della Ricerca di Pisa, 56124 Pisa, Italy; 7 Division of Translational Genomics, Exploratory Oncology Research and Clinical Trial Center, National Cancer Center, Kashiwa, 277-8575 Japan

**Keywords:** Non-small-cell lung cancer, Targeted therapies

## Abstract

**Background:**

Lung cancer cells overexpress mucin 1 (MUC1) and active subunit MUC1-CT. Although a peptide blocks MUC1 signalling, metabolites targeting MUC1 are not well studied. AICAR is a purine biosynthesis intermediate.

**Methods:**

Cell viability and apoptosis were measured in AICAR-treated *EGFR*-mutant and wild-type lung cells. AICAR-binding proteins were evaluated by in silico and thermal stability assays. Protein–protein interactions were visualised by dual-immunofluorescence staining and proximity ligation assay. AICAR-induced whole transcriptomic profile was determined by RNA sequencing. *EGFR*-*TL* transgenic mice-derived lung tissues were analysed for *MUC1* expression. Organoids and tumours from patients and transgenic mice were treated with AICAR alone or in combination with JAK and EGFR inhibitors to evaluate treatment effects.

**Results:**

AICAR reduced *EGFR*-mutant tumour cell growth by inducing DNA damage and apoptosis. MUC1 was one of the leading AICAR-binding and degrading proteins. AICAR negatively regulated JAK signalling and JAK1-MUC1-CT interaction. Activated EGFR upregulated MUC1-CT expression in *EGFR-TL*-induced lung tumour tissues. AICAR reduced *EGFR*-mutant cell line-derived tumour formation in vivo. Co-treating patient and transgenic mouse lung-tissue-derived tumour organoids with AICAR and JAK1 and EGFR inhibitors reduced their growth.

**Conclusions:**

AICAR represses the MUC1 activity in *EGFR*-mutant lung cancer, disrupting protein–protein interactions between MUC1-CT and JAK1 and EGFR.

## Introduction

Intermediate metabolites in the de novo purine biosynthesis usually promote benign and malignant cell proliferation [1, 2]. 5-aminoimidazole-4-carboxamide ribonucleoside (AICAR/acadesine) and adenosine analogue are intrinsic metabolites in the purine de novo biosynthesis pathway [3, 4]. The AICAR’s physiological expression level (300–1500 nM) is higher than adenosine (25–300 nM) [5–7]. However, higher levels of AICAR decrease tumour cell proliferation in leukaemias and other solid tumours [8–11]. In yeast cells, AICAR’s cytotoxicity is associated with the high concentration of its phosphorylated form [12]. In these conversions, adenosine kinase (ADK) catalyzes the phosphorylation reaction of AICAR and adenosine by adding phosphonate (-PO_3_H_2_) to form AICAR-PO_3_H_2_ (ZMP) and adenosine-PO_3_H_2_ (AMP), respectively [13]. As substrates of AMP-activated protein kinase (AMPK)—the energy homoeostasis regulator, the affinity of ZMP to AMPK is much weaker than AMP [14–17]. These data might provide clues to recent discoveries that AICAR’s anticancer roles are independent of AMPK, indicating that AICAR plays a prominent role in binding to other targets [18, 19]. Targeting lung cancer cells with AICAR in vitro and in vivo has demonstrated that AICAR is an effective anticancer molecule by our and other labs [20, 21]. It is still not well known how AICAR targets these lung cancer cells.

Mucin 1 (MUC1) is a transmembrane glycoprotein consisting of N-terminal alpha and C-terminal beta subunits (MUC1-CT) [22]. Previous studies have shown that the active subunit MUC1-CT is involved in tumorigenesis in lung and other cancers [23–28]. The protein–protein interaction study has demonstrated that epidermal growth factor receptor (EGFR) phosphorylates and activates MUC1-CT [29]. Upregulated MUC1-CT binds to and activates downstream effectors, such as signal transducer and activator of transcription 3 (STAT3), resulting in increased cell proliferation [30]. MUC1-CT has become a promising druggable target for treating cancer patients in preclinical models [31–33]. Even though the small molecule apigenin was reported to inhibit MUC1-CT dimerisation in the breast cancer cell lines, the inhibitory effect was probably mediated by blocking other targets [34–37]. Finding new small molecules directly blocking MUC1-CT will offer novel opportunities to treat MUC1-dependent lung tumours.

Approximately every 15 persons die of lung cancer in an hour in the US in 2022, accounting for 21% of all site cancer patients’ death hourly [38]. The death rate has dropped by three persons per hour compared to statistics in 2000 [39]. One reason for this decrease was the administration of widespread tyrosine kinase inhibitors (TKIs) that block mutant *EGFR* signalling as the first-line therapy in patients harbouring *EGFR* mutations [40–42]. In a preclinical setting, introducing mutant *EGFR* into mouse alveolar epithelial cells induces malignant phenotypes comparable to human lung adenocarcinoma. It demonstrates objective responses to EGFR induction or inactivation with changed protein expression for phosphorylated (p-EGFR) and total EGFR [43, 44]. Even though most patients respond well to these EGFR TKIs with prolonged survival, non-specific tissue distribution has limited their application. For example, osimertinib, the 3^rd^ generation of EGFR TKI targeting EGFR *T790M; L858R* (*TL*) mutations, is a cysteine-directed covalent drug that less-specifically binds to cysteine-rich tissues, including lung and spleen causing interstitial lung disease, diarrhoea, and hyperglycaemia [45, 46]. Consequently, the actual osimertinib concentration in lung tumours is below the effective dose by lysosomal sequestration with cysteine proteases, leading to acquired drug resistance [47, 48]. Compared to inactivating EGFR by osimertinib, degrading oncoprotein is becoming an alternative strategy for anticancer therapy that might decrease the incidence of drug resistance [49–51].

Besides the off-target effects, the long-term applications of EGFR TKIs have been limited due to alternative activation of inflammatory response such as Janus kinase (JAK)-STAT signalling [52]. Cytokine-induced activation of the JAK-STAT signalling pathway is associated with inflammatory diseases and lung tumour formation [53–55]. A recent study showed that blocking JAK-STAT signalling with the JAK inhibitors reduced tumour-promoting inflammation and tumour formation in the lungs [56, 57]. In addition, activation of JAK-STAT signalling causes de novo drug resistance to an irreversible EGFR TKI (afatinib) in lung cancer patients with *EGFR TL mutations* [58]. Thus, dually targeting JAK and EGFR with two inhibitors can better treat EGFR-dependent lung cancer [59].

In this study, our data has demonstrated that extrinsic AICAR treatment induces apoptosis and increases DNA damage in *EGFR*-mutant lung cancer cell lines. Mechanistically, AICAR binds and degrades the MUC1-CT protein. AICAR reduces JAK-STAT signalling by blocking physical protein–protein interactions between MUC1-CT and JAK1. AICAR treatment also reduces EGFR protein stability and activity as well as MUC1-CT expression. Clinically, higher expression of *MUC1* correlates with less overall and disease-free survival in lung adenocarcinoma patients at advanced stages. AICAR treatment inhibits cancer cell line-derived lung xenograft tumour growth in mice. AICAR treatment blocks 3D organoid growth from *EGFR*-mutant patient-derived xenograft and transgenic mouse lung tumour tissues. Combinational treatment with AICAR and EGFR and JAK inhibitors further decreases organoid formation. Collectively, our results highlight the new molecular mechanisms of AICAR that target MUC1-CT and its interactions with EGFR and JAK1 and provide a new therapeutic approach against *EGFR*-mutant lung cancer.

## Materials and methods

### Cell culture and cell lines

Human lung cancer cell lines with wild-type *EGFR* (H358, H23, A549, H441, H69, Calu-6, and H460) (ATCC) and with *EGFR* mutations (H1650, H1975, HCC827, PC9, PC9-ER, and H3255) (provided by Dr. Susumu Kobayashi) were cultured in DMEM (high glucose) (Gibco) with 10% fetal bovine serum (FBS), 2 mM L-glutamine and 1% penicillin–streptomycin. Primary lung fibroblast CCD-13Lu (ATCC) and rat alveolar macrophage NR8383 (ATCC) were cultured in DMEM (high glucose) (Gibco) with 10% FBS, 2 mM l-glutamine and 1% penicillin–streptomycin. Human lung microvascular endothelial cell HULEC-5A (ATCC) was cultured in MCDB131 (Gibco) supplemented with 10 ng/ml epidermal growth factor (EGF)(Gibco), 1 μg/ml hydrocortisone (Stemcell), 10 mM l-glutamine and 10% FBS. Immortalised tracheobronchial epithelial (AALE) cells were derived as previously described and maintained in SAGM media (Lonza) [60]. Cell line identities were confirmed by STR fingerprinting and all were found negative for mycoplasma using the MycoAler Kit (Lonza).

### 3D Organoid

For 3D organoid formation, single-cell suspensions (2000 cells/well/20 µl) were co-plated with geltrex (25 µl) in 96-well non-treated clear plates (Corning, Cat #08-772-53). The plate was incubated for 20 min at 37 °C, followed by adding 100 µl of complete growth media. The complete growth media was advanced DMEM/F12 with glutamax [1x], HEPES [1x], 1.25 mM N-Acetylcysteine, 10 mM Nicotinamide, 10 µM Forskolin, B27 [1x], 5 ng/ml Noggin, 100 ng/ml fibroblast growth factor 10 (FGF10), 20 ng/ml FGF2, 50 ng/ml EGF, 10 ng/ml platelet-derived growth factor A (PDGFA), 10 ng/ml FGF7, 1% penicillin–streptomycin and 10 µM Y-27632. Y-27632 was used only for the initial three days. The media was changed every three days for 24 days. The organoids were photographed with a microscope (Evos FL, Life Technology) and analysed by ImageJ.

### Patient-derived xenograft tumours specimens

The procedure for patient samples and data collection was conducted with approval by the Yale University Human Investigation Committee, and written informed consent was obtained from all patients. Tumour samples from one patient-derived xenograft (PDX) were generated at Yale Cancer Center as described previously [61, 62]. Briefly, tumour samples were digested in 1 mg/ml collagenase/dispase (Roche, Indianapolis, IN). After 3-h incubation, the cell viability was evaluated by trypan blue dye exclusion. Live single cells account for 90% of the whole population and dead cells account for less than 10%.

### Mouse models and transplantation

All animal research complied with protocols approved by the Institutional Animal Care and Use committees (IACUC) from BIDMC, Yale University, and UCF. *EGFR T790M-L858R* (*EGFR TL)/CCSP-rtTA* bi-transgenic mice and *tetO-Cre* transgenic mice were previously described [63]. To induce *EGFR TL* expression, 6-week-old female mice were fed a doxycycline (Dox) diet (Envigo) continuously for 0–14 weeks (EG0, EG1, EG2, EG10, EG14). Among these mice, one group fed a Dox diet for 8 weeks was followed by a regular diet for 2 weeks (EG8OFF2). The whole lung tissues were isolated at each time point for the following experiment. In lung tissues at EG14 that show apparent tumour nodules and normal tissue under a dissecting macroscope, the tumour nodules and normal lung tissues far (more than 0.5 cm) from the tumour nodules were isolated separately for gene expression analysis.

For tumour implantation, one million H1975 or A549 cells were co-injected with growth factor reduced Matrigel (BD) into 5-week-old female nude mice (NU/J) (Jackson Laboratory, Cat #002019) subcutaneously [64]. Fourteen mice were implanted for each lung cancer cell line. When tumour size reached 45 mm^3^, the mice were randomly grouped and treated with 300 mg/kg/day AICAR (MedchemExpress) in phosphate-buffered saline (PBS) (Sigma-Aldrich) or a vehicle (PBS) subcutaneously for 10 days (*n* = 7 per group). The mouse’s body weight was weighed with a scale, and tumour dimensions were measured with a caliper every two days. The tumour volume is calculated using a formula: volume = (length*width^2^)/2 as described previously [65]. At the endpoint of the experiment, the mice were euthanized, and the tumour and liver tissues were collected for imaging and staining. Investigators were blinded to the group allocation during the procedure.

### Antibodies

For cellular immunofluorescence staining and Duolink Proximity Ligation Assay, the primary mouse anti-human ZO-1 (1:100, Cat #339100) was from Thermo Fisher Scientific. Rabbit anti-MUC1-CT (1:500, Lot #GR181743-19, Cat #ab109185) was from Abcam and Armenian hamster anti-MUC1-CT (1:100, Lot #XI304891, Cat #MA511202) was from Thermo Fisher Scientific. Mouse anti-MUC1 (1:200, clone #VU4H5, Lot #1921, Cat #sc-7313) was from Santa Cruz. Rabbit anti-JAK1 (1:50, Lot #6, Cat #3344S) and rabbit anti-p-JAK1 (1:50, Lot #7, Cat #3331S) were from Cell Signalling. Secondary goat anti-rabbit IgG conjugated with Alexa Fluor 555 (1:500, Lot #UL292460, Cat #A32732), goat anti-mouse IgG conjugated with Alexa Fluor 555 (1:500, Lot #WH328965, Cat #A32727), and goat anti-rabbit IgG conjugated with Alexa Fluor 488 (1:500, Lot #WK341761, Cat #A32731TR) were from Life Technologies. The secondary goat anti-Armenian hamster conjugated with Alexa Fluor 555 (1:200, Lot #2530772, Cat #A78964) was from Life Technologies. For tissue immunofluorescence staining, rabbit anti-human Ki-67 (1:50, clone #SP6, Cat #MA5-14520) was from Thermo Fisher Scientific. Rabbit anti-γ-H_2_AX (S139) (1:50, Lot #6, Cat #7631) and rabbit anti-p21^Cip1^ (1:400, Lot #11, Cat #2947S) were from Cell Signalling.

For western blot, the primary polyclonal rabbit anti-H_2_AX antibody (1:1000, Lot #6, Cat #7631S), rabbit anti-p21^Cip1^ (1:1000, Lot #11, Cat #2947S), rabbit anti-γ-H_2_AX (S139) (1:1000, Lot #6, Cat #7631), rabbit anti-p-p53 (1:500, Lot #3, Cat #2526S), mouse anti-p53 (1:1000, Lot #3, Cat #18032S), rabbit anti-JAK1 (1:1000, Lot #6, Cat #3344S), rabbit anti-p-JAK1 (1:500, Lot #7, Cat #3331S), rabbit anti-TYK2 (1:1000, Lot #2, Cat #14193S), rabbit anti-p-TYK2 (1:500, Lot #2, Cat #9321S), rabbit anti-STAT3 (1:1000, Lot #6, Cat #12640S), mouse anti-p-STAT3 (1:500, Lot #9, Cat #9138 S), rabbit anti-EGFR (1:1000, Lot #24, Cat #4267S), and mouse anti-p-EGFR (Y1068) (1:1000, Lot #19, Cat #2236S) were from Cell Signalling. Mouse anti-PAICS antibody (1:1000, Lot #822101589, Cat #GTX83950) was from GeneTex. Rabbit anti-RBFA antibody (1:1000, Lot #QC57378-42788, Cat #ARP82050_P050), rabbit anti-LYSMD2 (1:1000, Lot #QC26841-091216, Cat #ARP55592_P050), and rabbit anti-SLC35C1 (1:1000, Lot #QC14138-20150217, Cat #ARP43989_T100) were from Aviva Systems. Mouse anti-ATIC antibody (1:1000, Lot #E0421, Cat #sc-365402) was from Santa Cruz. Rabbit anti-TMEM70 antibody (1:1000, Lot #0109940101, Cat #A13712), rabbit anti-TBXAS1 (1:1000, Lot #0011360101, Cat #A1988), rabbit anti-CYP39A1 (1:1000, Lot #5500004026, Cat #A20530), rabbit anti-CYP3A4 (1:1000, Lot #006270602, Cat #A14213), rabbit anti-PSME4 (1:1000, Lot #0059270101, Cat #A13815), rabbit anti-PIAS2 (1:1000, Lot #400001256, Cat #A5203), rabbit anti-ATP6V1D (1:1000, Lot #0061970201, Cat #A12940), rabbit anti-PRKAG1 (1:1000, Lot #0032670101, Cat #A7300), rabbit anti-PRKAG3 (1:1000, Lot #5500006732, Cat #A14132), rabbit anti-MCM5 (1:1000, Lot #4000001239, Cat #A5008), and rabbit anti-SEPTIN1 (1:1000, Lot #0128420101, Cat #A17471) were from ABclonal. Mouse anti-β-actin (1:10,000, clone C4, Lot #G0820, Cat #sc-47778) and mouse anti-GAPDH (1:10,000, Lot #C1721, Cat #47724) were from Santa Cruz and used as loading controls. Goat anti-rabbit IgG conjugated with horseradish peroxidase (HRP) (1:1000, Lot #WE327546, Cat #32460) and goat anti-mouse IgG conjugated with HRP (1:1000, Lot #WE321760, Cat #32430) were from Invitrogen and used as secondary antibodies.

### Compounds

AICAR was purchased from Selleck Chemicals (Lot #7B/237853, Cat #1802) for in vitro and MedchemExpress (Lot #97416, Cat #HY-13417) for in vivo applications. VX-509 (Lot #S754101, Cat #S7541), ABT-702 (Cat #S6619), and osimertinib (Cat #S7297) were purchased from Selleck Chemicals. Pierce protease and phosphatase inhibitor mini tablets (Lot #WD319834, Cat #A32959) were from Thermo Fisher Scientific and utilised for protein extraction.

### Compound treatment

AICAR, osimertinib, ABT-702, and VX-509 were reconstituted in sterile dimethyl sulfoxide (DMSO). The compound of interest was serially diluted in growth media for mono treatment to achieve the required concentration. For combination treatment, these compounds were serially diluted in growth media to achieve 2x or 3x solutions and combined to get the final 1x working solution.

### Cell viability assay

For 2D monolayer cell cultures, 3000 cells were plated into each well of a white 96-well plate in three replicates. After attachment, the cells were treated with AICAR, VX-509, osimertinib, and ABT-702 alone or in combinations for 72 h. The cell viability was measured at the treatment endpoint using the CellTiter-Glo® luminescent cell viability assay kit (Promega, Cat #G7570) according to the manufacturer’s instructions. The luminescent signal was recorded as relative light units (RLU). For 3D organoid cultures, the cell viability was measured using CellTiter-Glo 3D® Cell Viability Assay (Promega, Cat #G9681) according to the manufacturer’s protocol. The relative cell viability treated with the vehicle was normalised as 1.

### Cell apoptosis assay by flow cytometry

The eBioscience™ Annexin-V Apoptosis Detection Kit (APC) (Thermo Fisher Scientific) was used to measure apoptosis in lung cancer cells treated with AICAR. Briefly, the cells (H1975, PC9, H23, and A549) were plated in a 24-well plate and treated with AICAR (1 mM) for 0, 4, 7, and 16 h (H1975) or 7 h (PC9, H23, and A549). Then the cells were collected and incubated with Annexin-V conjugated with APC (1:40) in 200 µL binding buffer for 12 min at room temperature. Afterward, cells were stained with 7AAD. The fluorescence-labelled cells were measured with a CytoFLEX flow cytometer (Beckman Coulter) using APC (Ex/Em:633/660) and PI channels (546/647). 10,000 independent events were analysed by CytExpert software (Beckman Coulter).

### Cell immunofluorescence staining

Cells plated on 22-mm, 1.5-in thick poly-l-lysine-coated German coverslips (Neuvitro, Cat #GG-22-1.5-PLL) were fixed with 4% paraformaldehyde (Santa Cruz, Cat #30525-89-4), permeabilized with 0.1% Triton X-100 (Sigma-Aldrich, Cat #9002-93-1), and blocked with 5% bovine serum albumin (BSA) for 30 min at room temperature on a shaker. Followed by overnight incubation with rabbit anti-human MUC1-CT (Thermo Fisher Scientific), the cells were incubated with goat anti-rabbit IgG conjugated with Alexa Fluor 555 for 45 min at 37 °C. Cells were then counterstained with DAPI. For dual staining of p-JAK1 and MUC1-CT, the cells were co-incubated with Armenian hamster anti-MUC1-CT (Thermo Fisher Scientific) and rabbit anti-p-JAK1 (Cell Signalling) overnight at 4 °C. Followed by incubation with goat anti-Armenian hamster IgG conjugated with Alexa Fluor 555 (Thermo Fisher Scientific) and goat anti-rabbit IgG conjugated with Alexa Fluor 488 (Cell Signaling) at room temperature for 45 mins, the cells were counterstained with DAPI. Images were obtained with a Zeiss 710 confocal microscope.

### Duolink proximity ligation assay

H441 cells were plated on 22-mm, 1.5-in thick poly-l-lysine-coated German coverslips (Neuvitro) and treated with or without 1 mM AICAR for 1 h. Afterward, cells were fixed with 4% paraformaldehyde (Santa Cruz) for 10 mins at room temperature. The cells were then blocked using 1% BSA in TBS-T (Thermo Fisher Scientific, Cat #37520) for 30 min. After blocking, cells were incubated with mouse anti-ZO-1 primary antibody overnight followed by goat anti-mouse IgG conjugated with Alexa Fluor 488 (AF488) (Thermo Fisher Scientific, Cat #A11029) for the labelling cell membrane. The next day, cells were permeabilized using 0.1% Triton X-100 (Sigma-Aldrich) for 5 min at room temperature. Following that, cells were blocked using Duolink Blocking Buffer (Sigma-Aldrich, Cat #DUO82007) for 1 h at 37 °C in a pre-heated humidity chamber. The primary antibodies, anti-MUC1 (Santa Cruz, Cat #sc-7313) and anti-JAK1 (Cell Signaling, Cat #3344S) were diluted in the Duolink Antibody Diluent (Sigma-Aldrich, Cat #DUO82008) and incubated overnight at 4 °C. The following day, cells were washed in Duolink 1x Wash Buffer A (Sigma-Aldrich, Cat #DUO82047) for 5 min at room temperature. Proximity ligation assay (PLA) PLUS and MINUS probes conjugated with Cy3 (Sigma-Aldrich, Cat #DUO92004, DUO92002) were diluted 1:5 in the Duolink Antibody Diluent and applied to cells. They were incubated in a pre-heated humidity chamber for 1 h at 37 °C. For the ligation step, the 5x ligation buffer (Sigma-Aldrich, Cat #DUO82009) was diluted 1:5 in high-purity water and ligase (Sigma-Aldrich, Cat #DUO82029) was added in a 1:40 dilution. Cells were washed twice with 1x Wash Buffer A for 5 min each at room temperature. Then, cells were incubated with the ligation solution in a pre-heated humidity chamber for 30 min at 37 °C. For the amplification reaction, the 5X Amplification Buffer (Sigma-Aldrich, Cat #DUO82010) was diluted 1:5 in high-purity water and polymerase (Sigma-Aldrich, Cat #DUO82030) was added at a 1:80 dilution. Cells were washed twice for 5 min in 1x Wash Buffer A at room temperature. The amplification solution was added to the cells and incubated in a pre-heated humidity chamber for 100 min at 37 °C. After final incubation, cells were washed twice for 10 min each with 1x Wash Buffer B (Sigma-Aldrich, Cat #DUO82048) at room temperature, protected from light. Then the cells were washed for one min with a 0.01x Wash Buffer B at room temperature. Cells were mounted onto a glass slide using Duolink In Situ Mounting Media with DAPI (Sigma-Aldrich, Cat #DUO82040). Images were acquired using a Zeiss 710 confocal microscope using Zen Black programme. Images were quantified using ImageJ software (https://imagej.nih.gov/ij/, v1.53r, 21 April 2022).

### Plasmid transfection

To knock down or overexpress *MUC1* expression, 10,000 H1975 cells were transfected with shRNAs against *MUC1* (Origene, Cat #TL316625) or Lenti ORF clone of human *MUC1* (Origene, Cat #RC221340L4) in a 96-well plate. The sequences for four clones of sh-MUC1 are (TL316625A: ATATTAAGTTCAGGCCAGGATCTGTGGTG; TL316625B: AACGGAAGCAGCCTCTCGATATAACCTGA; TL316625C: ATCTCATTGCCTTGGCTGTCTGTCAGTGC; TL316625D: AGTGGCAGCCACTTCTGCCAACTTGTAGG). pGFP-C-shLenti shRNA Vector (TR30021) was used as scrambled control for shRNA, and pLenti-C-mGFP-P2A-Puro was used as a scrambled control for cDNA plasmid. The transfection procedure followed a standard protocol with PureFection (Systems Biosciences). After transfection, the cells were selected with 0.5 μg/ml puromycin for three days until mock-transfected cells were completely killed. Then the stable cell lines were used for downstream assays.

### RNA extraction and qRT-PCR

Total RNA was isolated from cells and tissues using mirVana miRNA Isolation Kit from Invitrogen (Ambion, Cat #AM1561) as described previously [66]. For mRNA expression analysis, a total of 500 ng RNA in each sample was input using a high-capacity RNA-to-cDNA kit according to the manufacturer’s instructions (Applied Biosystems, Cat #4387406). Real-time PCR was performed using TaqMan probes on Quant Studio Real-Time PCR (Thermo Fisher Scientific). Taqman gene expression probes included *MUC1* (Hs00159357_m1), *Egfr* (Mm01187858_m1), *Muc1* (Mm00449604_m1), *CTGF* (Hs00170014_m1), *ENO1* (Hs00361415_m1), and *PGM2* (Hs01055491_m1). *ACTB* (Hs01060665_g1), *GAPDH* (HsS02786624_g1), and *Gapdh* (Mm99999915_g1) were used as endogenous controls to analyse gene expression.

### High-throughput sequencing

The total RNA samples (1 µg) were processed by LC Sciences for RNA sequencing (RNA-seq). All RNA samples were analysed for quality on an Agilent 2100 Bioanalyzer. For mRNA-seq, AICAR-treated and parental H1975 cells (treated with 1 mM AICAR or vehicle for 4 h) were applied. The RNA samples were processed for RNA library generation using Illumina TruSeq RNA Sample Prep Kit (Cat #FC-122-1001). The subsequent sequencing was performed on the NovaSeq 6000 platform for 1 × 50-nt single-end sequencing, and the sequencing adaptor was trimmed from the raw reads. The reads are then mapped to the human genome (GRCh37) using Bowtie2 v2.2.9 [67]. The abundance was estimated using RSEM v1.3.0 [68], and the differential expression analyses were performed using EdgeR v3.12.1 [69]. Normalisation was done using the median ratio of the read count to the geometric mean of reading counts across samples as implemented in DESeq [70]. Genes showing significant differences (*p* < 0.05 and |log_2_FC|>1) were selected for enrichment analysis using GAGE v2.20.1 [71].

### Thermal stability assay

H1975 cells were plated in a 6-well plate at 80% confluency in duplicates and were treated with AICAR (1 mM) for 15 min. Cells were detached using a cell scraper (CellPro) and pelleted. The supernatant was removed, and the pellets were heated for three mins at their respective temperature (37–55 °C) in a mini dry bath incubator (Four E’s Scientific), followed by a three-min cool-down. Cell pellets were then resuspended in 80 μl of RIPA (radioimmunoprecipitation assay) buffer (Thermo Fisher Scientific) supplemented with protease and phosphatase inhibitor cocktail (Roche) to lyse the cells. The mixtures were shaken at 4 °C for 2 h, followed by centrifugation at 4 °C for 40 min at 14,000 RPM. The supernatants were collected and quantified, followed by western blotting.

### Western blotting

Cells were harvested and lysed with RIPA buffer supplemented with protease and phosphatase inhibitor cocktail (Roche). Protein concentrations of the extracts were measured using BCA assay (Pierce) and equalised with the extraction reagent. After normalisation, protein samples were separated via sodium dodecyl-sulfate polyacrylamide gel electrophoresis (SDS-PAGE) electrophoresis and transferred onto Immobilon-FL polyvinylidene fluoride membranes. The membranes were blocked with BSA for 1 h and then incubated overnight with primary antibodies in a cold room. Then the membranes were incubated with either goat anti-rabbit IgG conjugated with HRP (Invitrogen) or goat anti-mouse IgG conjugated with HRP (Invitrogen) for 1 h at room temperature. Followed by incubation in ECL Western Blotting Substrate (Pierce) for 5 min and visualised on ChemiDoc Imaging System (BioRad). The protein bands were quantified with ImageLab (BioRad).

### H&E and immunofluorescence staining

Samples were formalin-fixed, paraffin-embedded (FFPE), sectioned, and stained with hematoxylin-eosin (H&E) according to standard histopathological techniques. For immunofluorescence, tissues were heated to remove the paraffin and underwent antigen retrieval procedures through boiling with sodium citrate buffer. The sections were incubated with the following primary antibodies overnight at 4 °C: rabbit anti-human Ki-67 (1:50, Thermo Fisher Scientific), rabbit anti-p21^Cip1^ (1:400, Cell Signaling), and rabbit anti-γ-H_2_AX (1:50, Cell Signaling). Then the sections were incubated in secondary goat anti-rabbit Alexa Fluor 555 (Thermo Fisher Scientific) for 2 h at room temperature. Tissues were counterstained with DAPI and visualised using a Zeiss 710 confocal microscope. H&E images were captured on a microscope (Evos FL, Life Technology).

### Survival analysis

RNA-sequencing data of 506 lung adenocarcinoma (LUAD) patients in the Cancer Genome Atlas (TCGA) and their clinical information were downloaded from the Xena Public Data Hubs (https://xena.ucsc.edu) and cBioPortal (https://www.cbioportal.org/), respectively [72]. The log2(*x* + 1) transformed RSEM normalised counts were reported for gene expression levels [68]. The Kaplan–Meier survival curves were generated based on *MUC1* gene expression levels. The difference between groups was determined by the Log-rank test. Statistical comparisons were performed with a *t*-test (two groups) in all pertinent experiments.

### Statistical analyses

All experiments were performed in three to seven biological replicates and independently reproduced as indicated in figure legends unless described otherwise. Investigators were blinded to the group allocation during the procedure and data analysis. Data are presented as the means ± SEM. Unless otherwise stated, statistical significance was determined by a Student’s two-tailed *t*-test by GraphPad Prism (v8.4.3). *P* < 0.05 was considered statistically significant. Two-tailed *t*-test with Welch’s correction was applied for two samples with unequal variances. For three and more normally distributed samples, one-way ANOVA was used for multiple comparisons. For three and more normally distributed samples with unequal variances, the Brown-Forsythe and Welch ANOVA was used for multiple comparisons. Pearson correlation coefficient was used for correlation analysis between adenylosuccinate lyase (*ADSL)* and *MUC1* and other genes expression in 1,418 patient-derived lung tumour tissues from six independent datasets in the Lung Cancer Explorer web portal.

## Results

### AICAR increases cell death in lung cancer cells

**Fig. 1:**
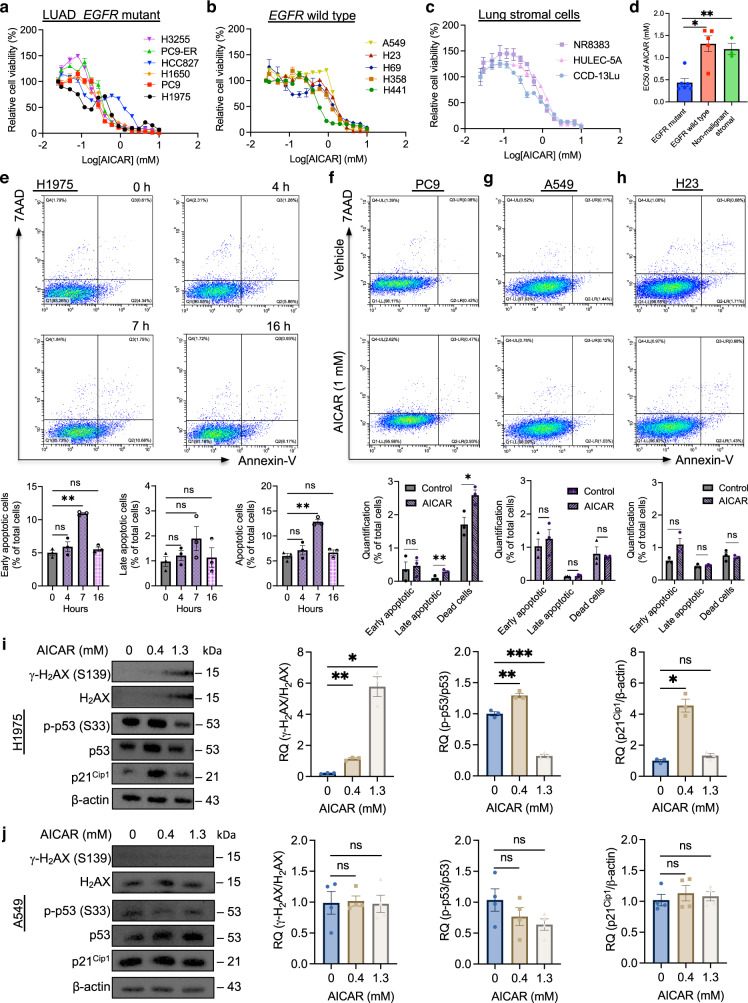
AICAR inhibits proliferation and induces apoptosis on *EGFR*-mutant lung cancer cell lines. **a** Cell viability assay of AICAR in *EGFR*-mutant lung adenocarcinoma cancer cell lines. 3000 cells (H3255, PC9-ER, HCC827, H1650, PC9, and H1975) were plated in a 96-well plate and treated with increasing doses of AICAR (0–10 mM). The cell viability was measured three days after treatment. Values were normalised to a vehicle-treated control group. *N* = 3 replicates. **b** Cell viability assay of AICAR in *EGFR* wild-type lung adenocarcinoma and small cell lung cancer cell lines. 3000 cells (A549, H23, H69, H358, and H441) were plated in a 96-well plate and treated with increasing doses of AICAR (0–10 mM). The cell viability was measured three days after treatment. Values were normalised to a vehicle-treated control group. *N* = 3 replicates. **c** Cell viability assay of AICAR in multiple types of primary lung stromal cells. 3000 cells (macrophages NR8383, endothelial cells HULEC-5A, and fibroblasts CCD-13Lu) were plated in a 96-well plate and treated with increasing doses of AICAR (0–10 mM). The cell viability was measured three days after treatment. Values were normalised to a vehicle-treated control group. *N* = 3 replicates. **d** Quantification of EC50 in *EGFR* mutant, wild-type, and non-malignant stromal lung cells. *N* = 3 replicates. **e** Flow cytometry analysis for time-dependent apoptosis in *EGFR*-mutant H1975 cells treated with AICAR. H1975 cells were plated in a 24-well plate and treated with AICAR (1 mM) for 0, 4, 7, and 16 h. After treatment, the apoptotic cells were quantified by flow cytometry analysis of cells stained with Annexin-V-APC and 7AAD. *N* = 4 replicates. **f**–**h** Flow cytometry analysis for apoptosis in multiple cell lines treated with AICAR. *EGFR*-mutant PC9 (**f**) and *EGFR* wild-type A549 (**g**) and H23 (**h**) cells were plated in a 24-well plate and treated with AICAR (1 mM) and vehicle control for 7 h. After treatment, the apoptotic cells were quantified by flow cytometry analysis of cells stained with Annexin-V-APC and 7AAD. *N* = 4 replicates. **i**, **j** Western blotting and relative quantification for expression levels of γ-H_2_AX (S139), H_2_AX, p-p53 (S33), p53, and p21^Cip1^ in H1975 and A549 cells treated with AICAR. The H1975 (**i**) and A549 (**j**) cells were treated with increasing doses of AICAR (0, 0.4, and 1.3 mM) for 22 h, followed by a western blot assay. β-actin, H_2_AX, and p53 were used as loading controls. *N* = 3 replicates. Data are mean ± s.e.m. and were analysed with one-way ANOVA (**d**) and Brown-Forsythe and Welch one-way ANOVA (**e**–**j**). *p < 0.05; **p < 0.01; ***p < 0.001; ns, not significant.

To explore the effects of AICAR treatment on lung cancer cell lines, we performed a cell viability assay using a panel of lung cancer cell lines based on the status of *EGFR* mutations (Table S1). AICAR treatment showed dose-dependent cytotoxic effects on cell viability across all tested cell lines harbouring *EGFR* mutations, including H1975, PC9, H3255, PC9-ER, H1650, and HCC827 cells (Fig. 1a). The EC50 (the concentration causing 50% treatment response) of AICAR in *EGFR*-mutant cell lines ranged from 0.298 mM to 0.88 mM (0.48±0.09 mM). To understand whether AICAR’s effects are dependent upon *EGFR* mutations, we tested drug response in another panel of lung cancer cells with wild-type *EGFR*, including H358, H23, H441, A549, and H69 (Fig. 1b). The average EC50 of tested *EGFR* wild-type cell lines (1.31 ± 0.18 mM) was higher than that of *EGFR*-mutant cell lines upon AICAR treatment (*p* < 0.05). To examine if there was any toxicity in stromal cells, we measured AICAR treatment response in a panel of primary lung stromal cell lines, including CCD-13Lu (fibroblasts), HULEC-5A (endothelial cells), and NR8383 (macrophages). Our data showed that AICAR caused less cytotoxicity in these stromal cells (EC50: 1.20 ± 0.13 mM) than in *EGFR*-mutant lung cancer cell lines (*p* < 0.01) (Fig. 1c, d). Therefore, these data suggest that *EGFR*-mutant lung tumour cells are sensitive to AICAR treatment.

To understand the mechanism of AICAR-induced cell toxicity, we measured cellular apoptosis by flow cytometry using annexin-V and 7-aminoactinomycin D (7AAD) staining in a panel of lung cancer cells treated with AICAR [73]. Our data showed that increased apoptosis rose highest 7 h and declined 16 h after AICAR treatment in *EGFR*-mutant H1975 cells, suggesting AICAR-induced cell apoptosis is time-dependent (Fig. 1e). Consistently, AICAR treatment increased late apoptosis and cell death in *EGFR*-mutant PC9 cells (Fig. 1f). However, cell apoptosis and death did not change significantly after AICAR treatment in *EGFR* wild-type cell lines, A549 and H23 (Fig. 1g, h). These data suggest that AICAR induces cytotoxicity by increasing cell apoptosis in *EGFR*-mutant lung cancer cells. Western blot assay demonstrated significant increases in phosphorylated H_2_AX (γ-H_2_AX), phosphorylated p53 (p-p53), and p21^Cip1^ in 0.4 mM AICAR-treated cells than in vehicle-treated cells, suggesting AICAR treatment increases DNA damage [74–76] (Fig. 1i). These data are consistent with a previous study showing AICAR-induced cell apoptosis in acute lymphoblastic leukaemia [10]. As expected, the expression levels of γ-H_2_AX, p-p53, and p21^Cip1^ did not change significantly in AICAR-treated A549 cells (Fig. 1j). Thus, our study suggests that AICAR induces lung tumour cell death through increasing DNA damage and cellular apoptosis in *EGFR*-mutant lung cancer cell lines.

### AICAR binds to and degrades MUC1-CT

ZMP is the toxic derivative of unphosphorylated AICAR through conversion by adenosine kinase reported in a previous study in yeast (Fig. S1A) [12]. It was not well understood if unphosphorylated AICAR could cause toxicity to human cells. To address this question, we blocked the change from AICAR to ZMP with an adenosine kinase inhibitor ABT-702 [77]. Our cell viability data showed that ABT-702 treatment alone did not show apparent cytotoxicity in H1975 cells, even at high concentrations (Fig. S1B). In 1 mM AICAR-treated cells, co-treating cells with ABT-702 at 0.5 μM and 5 μM significantly rescued AICAR-induced cell toxicity (*p* < 0.05 and *p* < 0.0001). However, in 3 mM AICAR-treated cells, 0.5 μM and 5 μM ABT-702 only rescued up to 10% of cell viability as compared with vehicle-treated cells (*p* < 0.001 and *p* < 0.001) (Fig. S1C). A synergistic assay showed that ABT-702 antagonised AICAR’s effect at 1 mM (*p* < 0.05) and 3 mM of AICAR (*p* < 0.05), in which the antagonism between ABT-702 and AICAR is less in 3 mM AICAR treatment than in 1 mM AICAR treatment (Fig. S1D). These data suggest that the cytotoxic effects of AICAR might be through phosphorylation-dependent and independent mechanisms.

**Fig. 2:**
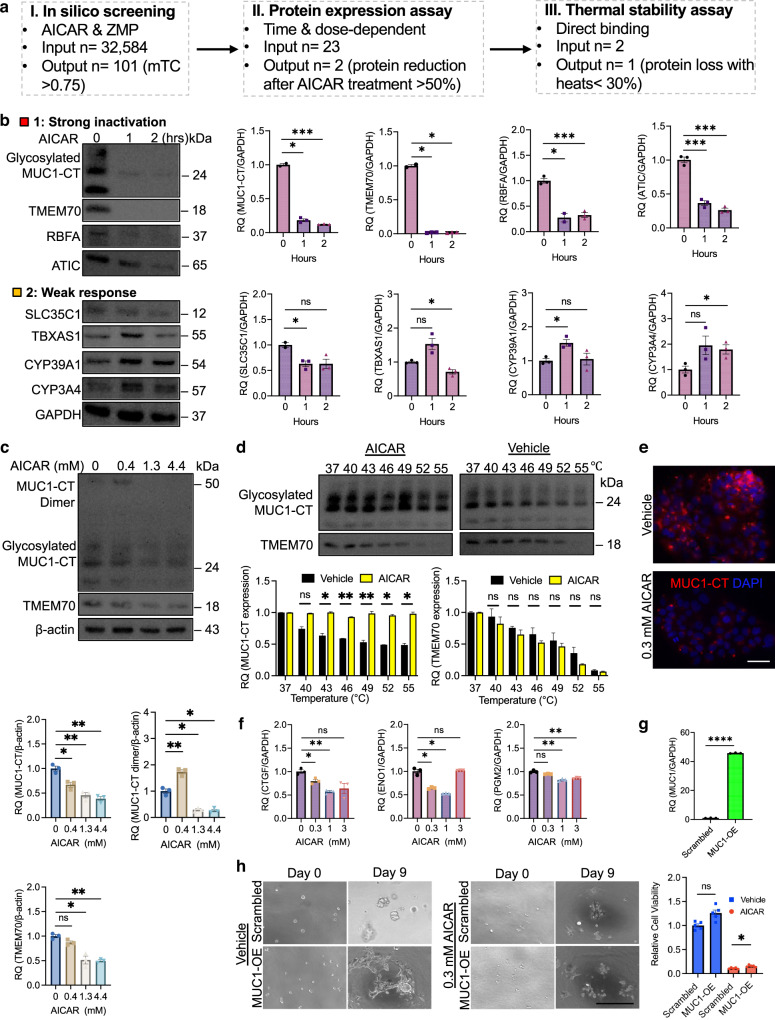
AICAR treatment reduces mucin 1-CT expression levels in lung cancer cells. **a** Diagram showing the strategy employed to shortlist AICAR-binding proteins. The three steps include in silico screening using FINDSITE^comb2.0^, protein expression assay, and thermal stability assay. **b** Time-dependent western blotting and relative quantification of protein expression for AICAR-binding proteins. The treatment responses on H1975 cells treated with 1 mM AICAR for 1 and 2 h were grouped by strong inactivation (type 1) and weak response (type 2). GAPDH was used as a loading control. *N* = 2–3 replicates. **c** Dose-dependent western blotting and relative quantification of protein expression for MUC1-CT and TMEM70. H1975 cells were treated with increasing doses of AICAR (0, 0.4, 1.3, and 4.4 mM) for 22 h, followed by a western blot assay. β-actin was used as a loading control. *N* = 3 replicates. **d** Thermal stability assay for MUC1-CT and TMEM70. H1975 cells were treated with 1 mM AICAR for 15 min. The cell pellets were heated for 3 min at their respective temperature (37–55 °C), followed by a western blot assay. *N* = 2 replicates. **e** Immunofluorescence staining for MUC1-CT in H441 cells. The cells were treated with 0.3 mM AICAR for 4 h and then incubated with rabbit anti-MUC1-CT primary antibody followed by goat anti-rabbit IgG conjugated with Alexa Fluor 555. The nucleus was counterstained with DAPI. The images were taken using a Keyence fluorescent microscope. Scale bar, 50 μm. **f** qRT-PCR analysis for MUC1-CT targeting genes. Expression levels for *CTGF*, *PGM2*, and *ENO1* were analysed by qRT-PCR in H1975 cells treated with 0.3 mM AICAR for 4 h. *GAPDH* was used as an endogenous control. *N* = 3 replicates. **g** qRT-PCR analysis for *MUC1* expression in H1975 cells with *MUC1* overexpression (OE). The cells were transfected with a lentiviral vector containing *MUC1* or scrambled control, followed by 0.5 µg/ml puromycin selection. The relative *MUC1* expression level in scrambled control cells was calibrated as 1. *GAPDH* was used as an endogenous control. *N* = 3 replicates. **h** Organoid formation assay for AICAR treatment response in H1975 cells overexpressing *MUC1*. The cells with *MUC1* overexpression or a scrambled control vector were plated at 2000 cells per well and treated with vehicle or 0.3 mM AICAR continuously for nine days. Images were taken with an EVOS microscope, and the treatment responses were quantified using a 3D Celltiter-Glo assay. *N* = 4–5 replicates. Scale bar, 300 µm. Data are mean ± s.e.m. and were analysed with Brown-Forsythe and Welch ANOVA (**b**, **c**, **f**, **h**); Welch’s *t*-test (**d**, **g**). *p < 0.05; **p < 0.01; ***p < 0.001; ns, not significant.

To find the new binding proteins of AICAR, we have applied a strategy using complementary approaches, including in silico screening, protein expression assay, and thermal stability assay (Fig. 2a). First, since AICAR’s function can be through its phosphorylated or unphosphorylated molecule, we analysed common targets bound by both AICAR and ZMP in the entire human proteome (*n* = 32,584) using a structure-based in silico tool, FINDSITE^comb2.0^ [78]. Among the top 101 binding proteins of AICAR and ZMP, 23 proteins were regulated by both AICAR and ZMP (Fig. S2, Tables S2 and S3). These putative binding targets included known AMPK subunit gamma-2 (PRKAG2) and ATIC (a component of de novo purine synthesising enzyme complex-forming purinosome) [16, 79, 80] (Fig. S2, Tables S2 and S3). Most targets have not been confirmed so far, including MUC1, phosphoribosylaminoimidazole carboxylase (PAICS)(another component of purinosome), minichromosome maintenance complex component 5 (MCM5), ribosomal RNA (rRNA) processing (ribosome binding factor A, RBFA), membrane transporters (solute carrier family 35 member C1 (SLC35C1), FXYD domain containing ion transport regulator 3 (FXYD3), ATPase H + transporting V1 subunit D (ATP6V1D)), proteasome activator subunit 4 (PSME4, a histone degradation marker in DNA damage), transmembrane protein 70 (TMEM70, a mitochondrial ATP synthase), and other candidates (LysM domain containing 2 (LYSMD2) and protocadherin alpha 5 (PCDHA5)) (Fig. S2). Besides these commonly shared targets by unphosphorylated and phosphorylated AICAR, the top ZMP-specific bound targets included AMPK subunits (PRKAG3 and PRKAG1) and IKAROS family zinc finger 5 (IKZF5, a transcription factor). The top acadesine-specific bound targets included thromboxane A synthase 1 (TBXAS1), cytochrome P450 family 39 subfamily A member 1 (CYP39A1), and cytochrome P450 family 3 subfamily A member 4 (CYP3A4) (Fig. S2). These data indicate the adenosine kinase-independent and dependent mechanism for AICAR-binding protein.

Next, to characterise top protein candidates targeted by AICAR, we performed an actual time-dependent protein expression screening by western blot assay, treating H1975 cells with 1 mM AICAR for 1 and 2 h. The data showed that AICAR treatment induced three responses: strong inactivation, weak response, and no response. In the AICAR type 1 response—strong inactivation (more than 50% reduction after 1- and 2-h treatment than untreated), 1 and 2-h AICAR treatment significantly decreased expression of MUC1-CT (*p* < 0.05 and *p* < 0.001), TMEM70 (*p* < 0.05 and *p* < 0.05), RBFA (*p* < 0.05 and *p* < 0.001), and ATIC (*p* < 0.001 and *p* < 0.001) (Fig. 2b). In the type 2 response—weak response (less than twofold change significantly), expression levels of SLC35C1 and TBXAS1 decreased significantly after 1-h (*p* < 0.05) and 2-h treatment (*p* < 0.05), and expression levels of CYP39A1 and CYP3A4 increased significantly after 1-h (*p* < 0.05) and 2-h treatment (*p* < 0.05) (Fig. 2b). In the type 3 response—no response (no significant change), the upregulation and downregulation of protein expression were not significantly changed after AICAR treatment, including PSME4, PIAS2, ATP6V1D, PRKAG1, PRKAG3, MCM5, septin 1 (SEPTIN1), PAICS, and LYSMD2 (Fig. S3). Thus, we focus on the top two candidates, MUC1-CT and TMEM70, for the subsequent studies.

Then a dose-dependent assay was used to examine if MUC1/MUC1-CT and TMEM70 were dysregulated in H1975 cells treated with increasing concentrations of AICAR (0, 0.4, 1.3, and 4.4 mM) for 22 h (Fig. 2c). We found that 0.4 mM AICAR treatment significantly decreased MUC1-CT expression compared to the vehicle-treated group (*p* < 0.05) (Fig. 2c). It was reported that MUC1 overexpression increases the build-up of MUC1-CT molecules forming homodimers in the cytoplasm and homodimer’s transport to the nucleus and mitochondria for signal transmission [81]. Our data showed that 1.3 mM and 4.4 mM AICAR treatment significantly reduced the expression levels of the MUC1-CT homodimer (*p* < 0.05 and *p* < 0.05), suggesting that AICAR might interfere with the function of MUC1-CT (Fig. 2c). However, expression levels of TMEM70 were not decreased significantly at a lower concentration (0.4 mM) of AICAR only at higher concentrations (1.3 mM and 4.4 mM) (Fig. 2c), indicating the effects of AICAR on protein stability of TMEM70 is less than MUC1-CT.

The physical binding of small molecules to targeted proteins yields a complex that can enhance protein stability compared with the free protein when protein is heated at increasing temperatures, as evidenced by the thermal stability assay [82]. We applied this assay for MUC1-CT and TMEM70. Our western blot data showed that AICAR incubation in H1975 cells significantly increased protein stability in MUC1-CT than vehicle-treated groups across 37–55 °C (Fig. 2d). In contrast, TMEM70 protein stability was not enhanced after AICAR treatment (Fig. 2d). Thus, these data have confirmed that MUC1/MUC1-CT is the topmost binding protein to AICAR.

To characterise the broad expression of MUC1 across cell lines, we analysed *MUC1* gene expression across 675 human cell lines from Genentech’s dataset [83]. H441 demonstrated the highest *MUC1* expression, and H1975 showed moderate expression in our panel of lung cell lines (Fig. S4A). Then we treated H441 cells with increasing doses of AICAR. Our data showed that only a high dose of AICAR treatment (3 mM) reduced MUC1-CT protein expression (*p* < 0.05) (fig. S4B), indicating that AICAR treatment can also block MUC1-CT expression in MUC1 highly abundant lung cancer cell lines.

To further visualise the subcellular localisation of MUC1-CT, we performed immunofluorescence staining for MUC1-CT in H441 cells. Our data showed that MUC1-CT is highly expressed, mainly localising at the cell membrane and cytoplasm (Fig. 2e). AICAR treatment dramatically decreased MUC1-CT expression in H441 cells, consistent with our western blot data (Fig. 2e). Previous studies showed that MUC1-CT could induce the expression of downstream targets, including connective tissue growth factor (*CTGF/CCN2*), enolase 1 (*ENO1*), and phosphoglucomutase 2 (*PGM2*) [84, 85]. To validate this, we performed a qRT-PCR analysis in H1975 cells and demonstrated decreased gene expression of *CTGF*, *PGM2*, and *ENO1* treated with various doses of AICAR (Fig. 2f). These data suggest that AICAR treatment degrades MUC1/MUC1-CT protein and decreases gene transcription of MUC1 downstream targets.

We then asked if changing the MUC1 expression level could regulate the cellular response to AICAR treatment. To answer this question, *MUC1* was overexpressed in H1975 cells with a lentiviral vector containing the *MUC1* gene. The *MUC1*-overexpressing cells showed a significant increase of *MUC1* compared to cells transfected with a scrambled vector (*p* < 0.0001) (Fig. 2g). *MUC1*-overexpressing H1975 cells demonstrated branch-like phenotypic changes but did not significantly increase 3D organoid formation than scrambled control cells in long-term culture (*p* > 0.05) (Fig. 2h). *MUC1*-overexpressing organoids were then continuously treated with 0.3 mM AICAR for nine days. Our data showed that MUC1 overexpression partially rescued AICAR-induced reduction in organoid formation (*p* < 0.05) (Fig. 2h). This suggests that MUC1 overexpression induces changes in cellular morphology and protects cells from AICAR-induced cell toxicity.

Since ADSL catalyzes the production of AICAR, we were curious about how AICAR impacts the ADSL expression [86]. Our western blot assay demonstrated that AICAR treatment decreased the expression levels of ADSL (*p* < 0.05) (Fig. S5A), suggesting negative feedback of AICAR on ADSL expression levels. Since MUC1 is bound directly by AICAR, we asked if the MUC1 expression level is correlated to ADSL. Our western blot data has shown the increased expression of MUC1-CT and decreased expression of ADSL in lung cancer cell lines H1975 and H1650 than in a non-tumorigenic and immortalised lung epithelial cell line AALE (Fig. S5B), indicating a negative association between MUC1-CT and ADSL expression. To further examine if MUC1 can affect ADSL expression, we overexpressed *MUC1* in H1975 cells with an 18.3-fold increase at protein levels by western blotting (*p* < 0.05) (Fig. S5C). As expected, the ADSL expression level decreased by 55% in MUC1-overexpressing cells, suggesting MUC1 inhibits ADSL protein expression (*p* < 0.05) (Fig. S5C). To investigate the association between *ADSL* and *MUC1* at mRNA levels, we performed a correlation analysis among ADSL and AICAR-binding candidates including MUC1 in six independent datasets consisting of human lung cancer tissues from patients (*n* = 1418) using Lung Cancer Explorer [72, 87–92]. Our analysis demonstrated that *MUC1* was the top gene showing a negative correlation with *ADSL* expression (*r* = –0.47, *p* < 0.00001) (Fig. S5D and Table S4). Collectively, these data suggest that MUC1 negatively regulates ADSL expression.

To our knowledge, there are no reports of AICAR targeting MUC1. Therefore, our study focused on MUC1 and the signalling molecule MUC1-CT.

### AICAR inhibits JAK1-MUC1 protein–protein interaction

**Fig. 3:**
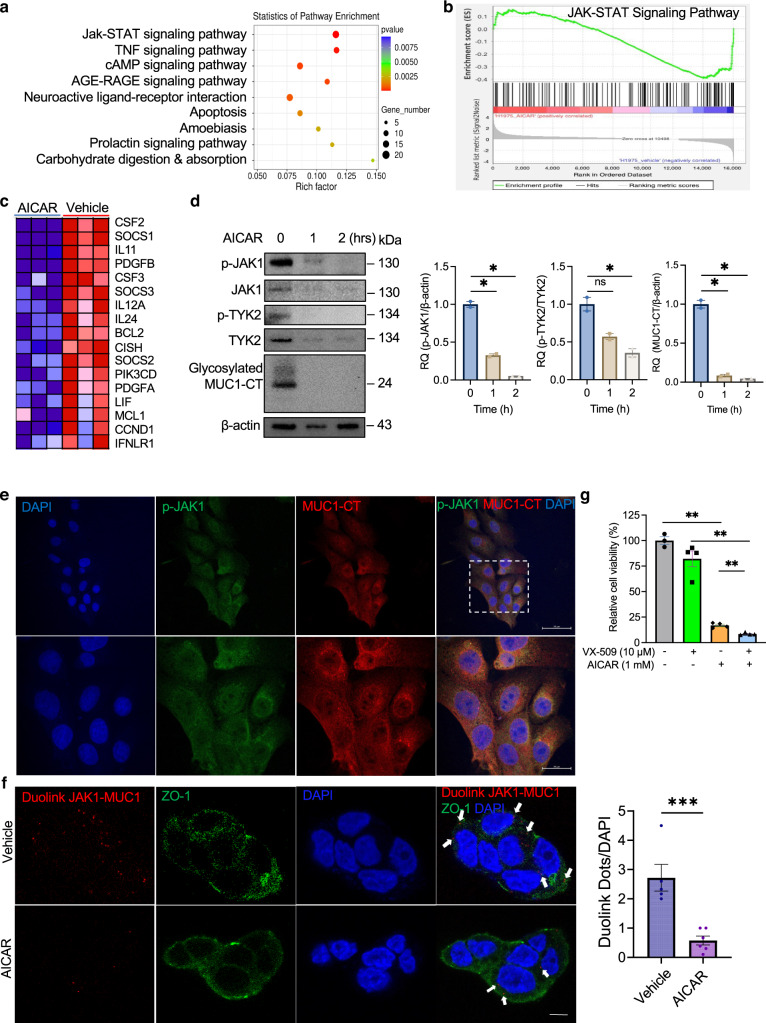
AICAR blocks protein–protein interactions between MUC1 and JAK1. **a** Top KEGG signalling pathways differentially expressed in H1975 cells treated with AICAR. The cells were treated with 1 mM AICAR for 4 h, followed by whole transcriptomic analysis. *N* = 3 replicates. **b** Enrichment plot by gene set enrichment analysis for the JAK-STAT signalling pathway in H1975 cells treated with AICAR. Profile of the running enrichment score (ES) (top) and positions of gene set members on the rank-ordered list (bottom) were shown. *N* = 3 replicates. **c** A heat map showing top enriched genes of the JAK-STAT signalling pathway in H1975 cells treated with AICAR compared with vehicle-treated cells. **d** Longitudinal analysis of p-JAK1, p-TYK2, and MUC1-CT expression. H1975 cells were treated with 1 mM AICAR for 0, 1, and 2 h followed by a western blot assay. β-actin was used as a loading control. *N* = 3 replicates. **e** Confocal images for co-localisation of MUC1-CT and p-JAK1 in lung cancer cells. H1975 cells were co-incubated with Armenian hamster anti-MUC1-CT and rabbit anti-p-JAK1 primary antibodies for 2 h. Then the cells were incubated with secondary antibodies conjugated with Alexa Fluor 488 or 555. The nucleus was counterstained with DAPI. The images (top: lower magnification; bottom: higher magnification) were taken using a Zeiss confocal fluorescent microscope. Scale Bar, 50 µm (top) and 20 µm (bottom). **f** Duolink ligation assay and confocal imaging for physical MUC1-JAK1 interactions. H441 cells were treated with vehicle or 1 mM AICAR for 1 h. After treatment, the cells were incubated with mouse anti-ZO-1 primary antibody overnight, followed by anti-mouse IgG conjugated with Alexa Fluor 488. Then the cells were co-incubated with anti-MUC1 and anti-JAK1 primary antibodies, followed by incubation with proximity ligation assay probes conjugated with Cy3, ligation, and amplification steps. The nucleus was counterstained with DAPI. The images were taken using a Zeiss confocal fluorescent microscope, and the Duolink dots were quantified using Image J. Scale Bar, 20 µm. **g** Cell viability assay of H1975 cells treated with AICAR and VX-509. Cells were plated in a 96-well plate and treated with AICAR (1 mM) with or without VX-509 (10 μM). The cell viability was measured three days after treatment. Values were normalised to a vehicle-treated control group. *N* = 3–4 replicates. Data are mean ± s.e.m. and were analysed with Welch’s *t*-test (**a**, **b**, **f**); Brown-Forsythe and Welch one-way ANOVA (**d**, **g**). *p < 0.05; **p < 0.01; ***p < 0.001; ns, not significant.

To define the molecular mechanisms of targeting tumour cells by AICAR, we treated H1975 cells with 1 mM AICAR for 4 h and performed an unbiased whole transcriptome assay. Analysis of differentially expressed genes (Table S5) showed that the top significantly dysregulated KEGG signalling pathways were the JAK-STAT signalling pathway (*p* = 2.90E-06), tumour necrosis factor (TNF) signalling pathway (*p* = 5.04E-05), adenosine 3’,5’-cyclic monophosphate (cAMP) signalling pathway (*p* = 3.38E-04), advanced glycation end products-receptor for advanced glycation end products (AGE-RAGE) signalling pathway in diabetic complications (*p* = 3.83E-06), neuroactive ligand–receptor interaction (*p* = 6.42E-04), and apoptosis (*p* = 1.46E-03) (Fig. 3a and Table S6). The gene set enrichment analysis (GSEA) showed that the JAK-STAT signalling pathway was negatively correlated to AICAR treatment (normalised enrichment score = –1.67, *p* < 1.0E-09) (Fig. 3b and Table S7). Among this signalling pathway, these downregulated genes were cytokines (*CSF2, CSF3, IL11, IL6, IL5, IL17D*), growth factors (*LIF, PDGFA, PDGFB*), hormones (*CSH1, CSH2, THPO*), receptors (*EGFR, EPOR, IFNLR1, IL7R, IL4R, IL27RA, PIK3R1*), and STAT effectors related to anti-apoptosis and cell-cycle progression (*SOCS1, SOCS2, SOCS3, BCL2, MCL1, BCL2L1, CCND1, MYC*) (Fig. 3c, Fig. S6 and Table S8). These data suggest that AICAR interferes with JAK-STAT signalling.

The JAK-STAT signalling pathway is dysregulated through the JAK family in the cancers [93]. To determine how AICAR interacts with the JAK-STAT signalling pathway, we performed western blotting for phosphorylated JAK1 (p-JAK1), p-TYK2, and MUC1-CT in H1975 cells treated with AICAR. Our data demonstrated that AICAR treatment for 1 and 2 h concurrently decreased expression levels for p-JAK1 (*p* < 0.05 and *p* < 0.05), p-TYK2 (*p* > 0.05 and *p* < 0.05) and MUC1-CT (*p* < 0.05 and *p* < 0.05) comparing with the vehicle-treated cells (Fig. 3d). These data indicate that MUC1 might interact with JAK1 and mediate AICAR’s effects. To validate this hypothesis, we performed dual-immunofluorescence staining for MUC1-CT and p-JAK1 to examine whether these two proteins co-localise in lung cancer cells. As expected, our confocal microscopic data showed that MUC1-CT and p-JAK1 are co-localised in the cytoplasm of tumour cells, predominately close to the nucleus (Fig. 3e). Then we asked if JAK1 interacts with MUC1 physically and if AICAR can interfere with this interaction. Utilising the Duolink proximity ligation assay that allows in situ visualising of direct protein–protein interaction evidenced by proximity signals with Duolink immunofluorescent dots [94], we found that the Duolink dots were shown in cytoplasm close to the nucleus, which is consistent to our dual-immunofluorescence data. As expected, the proximity signals in AICAR-treated cells were 4.7-fold less than those in the vehicle-treated cells (*p* < 0.001), suggesting that JAK1 interacts with MUC1 directly and that AICAR treatment dampens this interaction (Fig. 3f).

Since AICAR deactivates JAK signalling, we asked if AICAR could enhance the effects of small molecule inhibitors against JAK. We applied a pan-JAK inhibitor VX-509 with AICAR into H1975 cells and examined cell treatment response to address this question [95]. Our WB data showed that VX-509 significantly decreased expression levels of JAK1 (*p* < 0.05) and p-STAT3 (*p* < 0.05), confirming VX-509’s roles in blocking JAK signalling (Fig. S7). Next, the cell viability assay demonstrated that VX-509 alone did not induce apparent cell toxicity (Fig. 3g). VX-509 (10 μM) combined with AICAR (1 mM) significantly reduced cell viability compared with VX-509 or AICAR treatment alone (*p* < 0.01 and *p* < 0.01) (Fig. 3g). These data suggest that AICAR can functionally promote the roles of pan-JAK1 inhibitors in cancer cells.

### AICAR blocks mutant *EGFR*-induced MUC1 activation

**Fig. 4:**
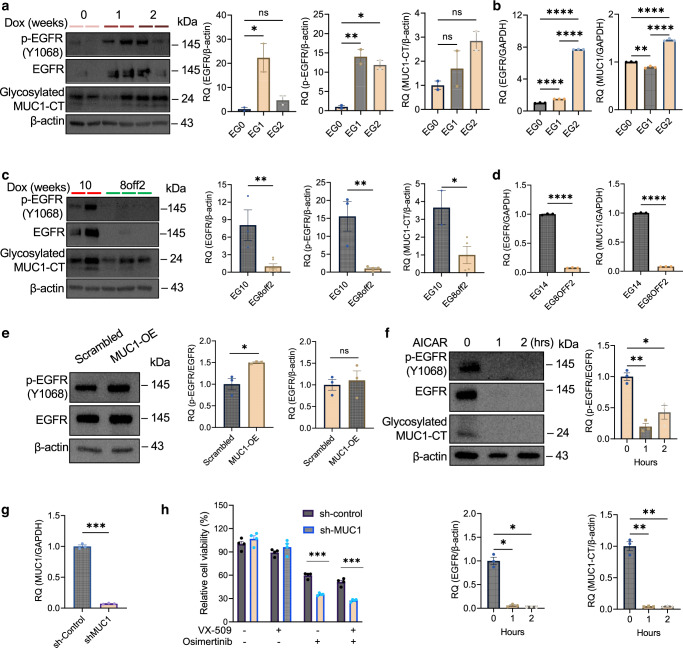
Activated EGFR upregulates MUC1-CT expression. **a** Western blotting and quantitative analysis for p-EGFR (Y1068), EGFR, and MUC1-CT in *EGFR TL* (*T790M; L858R*)-induced lung tissues from transgenic mice. The mice were fed doxycycline (Dox)-impregnated food pellets for 0, 1, and 2 weeks followed by whole lung-tissue extraction. *N* = 2 replicates. **b** qRT-PCR analysis of gene expression for *EGFR* and *MUC1* in *EGFR TL*-induced lung tissues. *Gapdh* was used as an endogenous control. EG0, EG1, and EG2 represent tissues from the mice fed with dox-impregnated food pellets for 0, 1, and 2 weeks, respectively. *N* = 3 replicates. **c** Western blotting and quantitative analysis for p-EGFR (Y1068), EGFR, and MUC1-CT in *EGFR TL*-induced lung tissues after EGFR inactivation. The mice fed with Dox-impregnated food pellets for 8 weeks were given either the same Dox diet for an additional 2 weeks (EG10) or a regular diet for 2 weeks (EG8off2). Then the whole lung tissues were extracted for protein expression assay. *N* = 2 replicates. **d** qRT-PCR analysis of gene expression for *EGFR* and *MUC1* in mouse *EGFR TL*-induced lung tissues after EGFR inactivation. The lung tissues from EG14 and EG8OFF2 mice were extracted for RNA analysis. *GAPDH* was used as an endogenous control. *N* = 3 replicates. **e** Western blotting and quantitative analysis for p-EGFR (Y1068) and EGFR expression in H1975 cells with *MUC1* overexpression (OE). β-actin was used as a loading control. *N* = 3 replicates. **f** Western blotting and quantitative analysis for p-EGFR (Y1068), EGFR, and MUC1-CT expression in H1975 cells treated with 1 mM AICAR for one and 2 h. β-actin was used as a loading control. *N* = 3 replicates. **g** qRT-PCR analysis for *MUC1* gene expression in H1975 cells with *MUC1* knockdown. The cells were transfected with a lentiviral vector containing shRNA against *MUC1* (shMUC1) or a scrambled control vector (sh-Control), followed by a 0.5 µg/ml puromycin selection. *GAPDH* was used as an endogenous control. *N* = 3 replicates. **h** Cell viability assay of H1975 cells treated with osimertinib and VX-509. 3000 cells with *MUC1* knockdown (sh-MUC1) and a negative control vector (sh-control) were plated in a 96-well plate and treated with VX-509 (10 μM), osimertinib (0.5 μM), or both. The cell viability was measured three days after treatment. Values were normalised to a vehicle-treated sh-control group. *N* = 4 replicates. Data are mean ± s.e.m. and were analysed with unpaired two-tailed *t*-test (**c**, **d**, **e**, **g**); one-way ANOVA (**a**, **b**); Brown-Forsythe and Welch ANOVA (**f**, **h**). *p < 0.05; **p < 0.01; ***p < 0.001; ****p < 0.0001; ns, not significant.

A previous study showed that transgenic MUC1 physically activates wild-type *EGFR* in the mouse mammary gland [96]. We were curious if oncogenic *EGFR* interacts with MUC1-CT in a transgenic lung cancer mouse model. To address these questions, we collected whole lung tissues from *EGFR* dual-mutation (*T790M; L858R, TL*)-driven and doxycycline (Dox)-inducible transgenic mice. Our western blot analysis demonstrated that MUC1-CT expression rose concurrently with p-EGFR and EGFR 1- and 2 weeks post-administration of Dox (Fig. 4a). Consistently, gene expression levels for *EGFR* (*p* < 0.0001 and *p* < 0.0001) and *MUC1* (*p* < 0.01 and *p* < 0.0001) were upregulated significantly in lung tissues 1 and 2 weeks upon Dox induction (Fig. 4b). In contrast, comparing to Dox-induced lung tumour tissues continuously for 10 weeks (EG10), withdrawing Dox for 2 weeks after 8-week Dox induction (EG8off2) in mice significantly inhibited protein expression for p-EGFR (*p* < 0.01), EGFR (*p* < 0.01), and MUC1-CT (*p* < 0.05) (Fig. 4c). Similarly, gene expression levels for *EGFR* (*p* < 0.0001) and *MUC1* (*p* < 0.0001) decreased significantly in lung tissues from EGF8OFF2 compared to those from EG14 (Fig. 4d). Collectively, these data suggest that mutant *EGFR TL* induces MUC1/MUC1-CT expression in lung cancer tissues.

We then asked if *MUC1* could regulate EGFR activity. To test this hypothesis, we assessed protein levels for p-EGFR and EGFR in H1975 cells overexpressing *MUC1*. Our western blot data demonstrated that overexpressing MUC1 significantly increased the p-EGFR expression by 1.5-fold (*p* < 0.05) and did not change EGFR expression levels significantly (Fig. 4e). These data suggest that MUC1 overexpression positively regulates EGFR phosphorylation in *EGFR*-mutant lung cancer.

Since MUC1 and EGFR regulate each other mutually, we asked if AICAR treatment would reduce both expression levels. In H1975 cells treated with AICAR for 1 and 2 h, expression levels for p-EGFR, EGFR, and MUC1-CT decreased significantly (Fig. 4f). A previous study showed that lung tumour cells are featured with high EGFR protein stability [50]. Our data suggest that AICAR not only inactivates MUC1-CT and EGFR activity but may also degrade EGFR protein stability, thus providing a new strategy to block EGFR-driven oncogenesis.

We then asked if simultaneously targeting MUC1 and EGFR could improve lung cancer treatment. We applied a vector containing shRNAs against *MUC1* into H1975 cells by gene transfection. qRT-PCR analysis showed that *MUC1* gene expression levels decreased by 93% after knocking down *MUC1* (*p* < 0.001) (Fig. 4g). We then treated H1975 cells with *MUC1* knocked down with or without 0.5 μM osimertinib, a third-generation EGFR TKI that inhibits dual *EGFR* mutations [97, 98]. The cells with *MUC1* knockdown were more sensitive to osimertinib, evidenced by a further reduction of cell viability than in the scrambled control cells in short-term 2D culture (*p* < 0.001) (Fig. 4h). These data confirmed our hypothesis that dually targeting both MUC1 and EGFR could better inhibit the growth of tumour cells. Furthermore, to understand if blocking the JAK signalling pathway can further enhance the above effects, we co-treated cells with the scrambled control and *MUC1* knockdown together with 10 μM VX-509 and 0.5 μM osimertinib. Our data showed that combination treatment with VX-509 and osimertinib further reduced cell viability than cells with scrambled control (*p* < 0.001) (Fig. 4h).

Thus, our data suggest that EGFR positively interacts with MUC1, and AICAR treatment blocks these interactions.

### AICAR blocks *EGFR*-mutant tumour formation in vivo

**Fig. 5:**
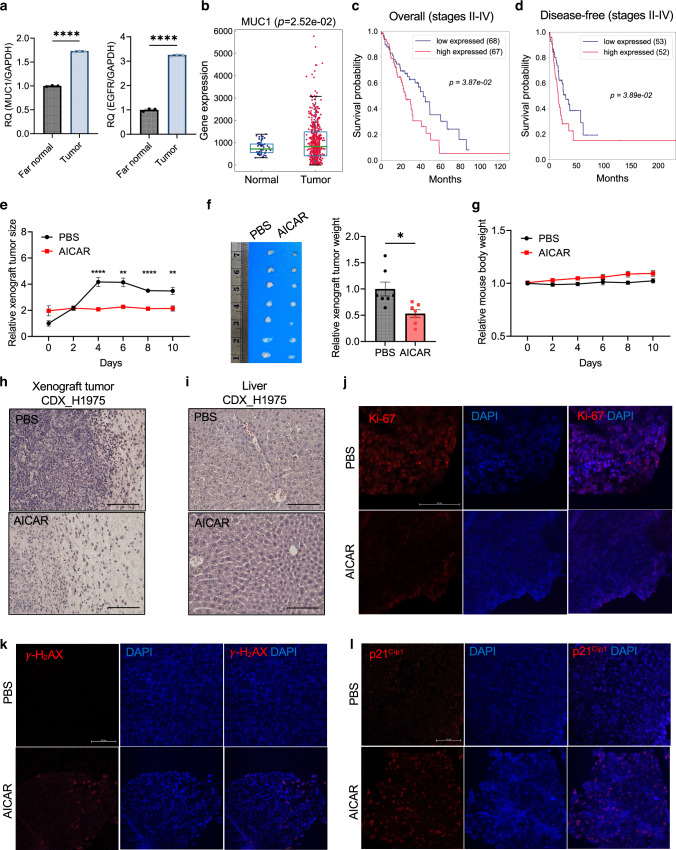
AICAR blocks *EGFR*-mutant tumour formation in mice. **a** qRT-PCR analysis of gene expression for *EGFR* and *MUC1* in *EGFR TL*-induced lung tissues from transgenic mice. The far normal and tumour tissues from transgenic mice 14 weeks after Dox induction were extracted for RNA analysis. *Gapdh* was used as an endogenous control. *N* = 3 replicates. **b** Differential *MUC1* gene expression in lung adenocarcinoma (LUAD) compared with tumour-adjacent tissues by analysing the TCGA_LUAD dataset. *N* = 59 (normal), and *N* = 517 (tumour). **c** Patients’ overall survival in lung adenocarcinoma patients at stages II–IV. The median expression levels of *MUC1* were used for a cut-off of high and low expression for *MUC1*. *N* = 135. **d** Patients’ disease-free survival in lung adenocarcinoma patients at stages II–IV. The median expression levels of *MUC1* were used for a cut-off of high and low expression for *MUC1*. *N* = 105. **e** Xenograft tumour growth in mice treated with AICAR. The xenograft tumour was pre-established by implanting 1 million H1975 cells subcutaneously. When the tumour reached 45 mm^3^, the mice were treated with 300 mg/kg/day AICAR in PBS or a vehicle subcutaneously for ten days. The tumour was measured with a digital caliper, and the tumour size was calculated. *N* = 7 replicates. **f** Xenograft tumour images and relative weight quantification from mice treated with AICAR or PBS. The average tumour weight from the PBS-treated group is normalised as 1. *N* = 7 replicates. **g** Mouse body weight after treatment with AICAR or PBS for ten days. *N* = 7 replicates. **h**, **i** H&E staining of subcutaneous tumours (**h**) and liver tissues (**I**) from H1975 cell line-derived xenograft (CDX) treated with PBS or AICAR. Scale bar, 125 μm. **j**–**l** Immunofluorescence staining for Ki-67 (**j**), γ-H_2_AX (**k**), and p21^Cip1^ (**l**) in subcutaneous tumours from H1975 CDX treated with PBS or AICAR. Scale bar, 100 μm. Data are mean ± s.e.m. and were analysed with unpaired two-tailed *t*-test (**a**, **b**); log-rank test (**c**, **d**); Welch’s *t*-test (**e**–**g**). *p < 0.05; **p < 0.01; ****p < 0.0001; ns, not significant.

To determine the differential *MUC1* expression between malignant and non-malignant lung tissues, we isolated the tumour nodules and normal lung tissues far (more than 0.5 cm) from visible tumour nodules from the *EGFR TL*-induced lung cancer mice fed Dox for 14 weeks. Our qRT-PCR analysis showed that *MUC1* expression levels in the tumour tissues were 1.7-fold higher than that in the tumour-far normal tissues (*p* < 0.0001), consistent with upregulation of *EGFR* gene expression in the same tumour tissues (*p* < 0.0001) (Fig. 5a). Then we performed a transcriptomic analysis of lung tumour and normal tissues from patients in the TCGA dataset to examine the clinical impact of *MUC1* expression. In cancer patients, *MUC1* gene expression is significantly higher in lung adenocarcinoma than in normal lung tissues (*p* = 2.52e-02) (Fig. 5b). These data suggest that *MUC1* expression is upregulated in lung adenocarcinoma tissues. Further survival analysis showed that higher *MUC1* expression levels were correlated with poorer overall survival (*p* = 3.87e-02) and disease-free progression (*p* = 3.89e-02) in the advanced (II–IV) stages but not in stage I patients (Fig. 5c, d and Fig. S8A, B). These data suggest that *MUC1* expression negatively correlates with lung cancer patients’ prognosis at advanced stages.

To evaluate AICAR’s anticancer treatment in vivo, we transplanted 1 million H1975 cells into mice subcutaneously to establish cell line-derived xenograft (CDX) tumours (*n* = 7). When xenograft tumours reached 45 mm^3^, the mice were injected with 300 mg/kg/day AICAR subcutaneously for 10 days. Compared with PBS-treated groups, AICAR treatment significantly reduced the tumour size from day 4 to day 10 (*p* < 0.01) (Fig. 5e). The tumour weight was reduced by 47% in AICAR-treated mice compared with PBS-treated mice (*p* < 0.05) (Fig. 5f). As expected, AICAR treatment did not induce significant loss of body weight during the period of treatment (*p* > 0.05) (Fig. 5g). The H&E staining data showed decreased tumour cells with clear and continuous boundaries in tumour tissues from AICAR-treated mice than those from PBS-treated mice (Fig. 5h). The liver tissues stained by H&E did not show noticeable phenotypic changes between groups treated with AICAR and PBS (Fig. 5i). To examine molecular profiles for proliferation and DNA damage, we performed immunofluorescence staining in tissues from mice treated with AICAR. Our data displayed that the proliferative marker Ki-67 expression levels decreased in AICAR-treated groups, suggesting reduced cell proliferation after AICAR treatment in tumours (Fig. 5j). In contrast, the expression levels of γ-H_2_AX and p21^Cip1^ increased after AICAR treatment, suggesting increased DNA damage in tumour cells after AICAR treatment (Fig. 5k, l). Thus, our data suggest that AICAR blocks H1975-derived xenograft tumour growth in vivo by decreasing proliferation and increasing DNA damage in tumour cells.

Then we queried if AICAR could also target *EGFR* wild-type lung cancer cell-derived tumours. To address this question, we transplanted 1 million A549 cells subcutaneously into mice and administrated 300 mg/kg/day AICAR or PBS for 10 days when tumour sizes reached 45 mm^3^ (n = 7). In contrast to H1975-derived xenografts, the tumour size and weight of A549-derived xenografts did not decrease significantly after AICAR treatment (Fig. S9a–c). The mouse’s body weight did not change significantly during treatment (Fig. S9d). Microscopic phenotypes for xenograft tumours and liver tissues by H&E staining demonstrated no apparent changes after AICAR treatment (Fig. S9e, f). These data suggest that mice harbouring A549-derived xenograft tumours do not respond to AICAR treatment, unlike those with H1975-derived xenografts.

### Co-targeting EGFR and JAK with AICAR reduce organoid growth from PDX and transgenic mouse tumour

**Fig. 6:**
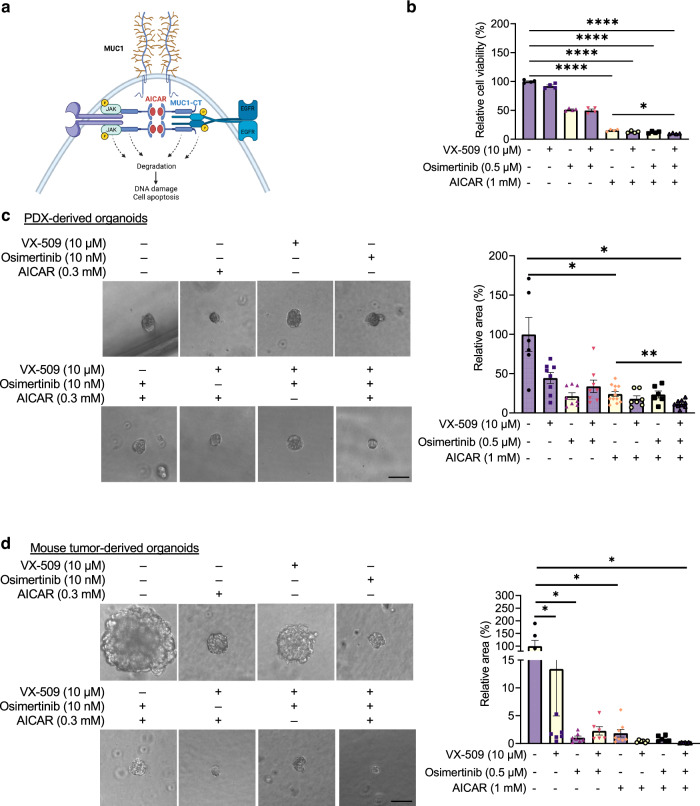
AICAR combined with osimertinib and VX-509 block 3D structure formation in patient and transgenic mouse-derived tumours. **a** A diagram showing mechanisms of AICAR’s anticancer roles. In MUC1-dependent tumours, AICAR treatment directly binds and degrades MUC1-CT, increasing DNA damage in tumour cells. The degraded MUC1-CT de-stabilises p-EGFR and p-JAK1, further inactivating tumour-supportive signals. Created with BioRender.com. **b** Treatment response to VX-509 and osimertinib and AICAR in H1975 cells. 3000 cells were plated in a 96-well plate and treated with VX-509 (10 μM), osimertinib (0.5 μM), AICAR (1 mM), or a combination. The cell viability was measured 3 days after treatment. Values were normalised to a vehicle-treated group. *N* = 4 replicates. **c**, **d** Growth of PDX (**c**) and transgenic mouse *EGFR TL*-induced lung tumour (**d**)-derived organoids treated with AICAR, osimertinib, and VX-509. 2000 cells were plated in organoid-culture media followed by treatments with AICAR (1 mM), osimertinib (0.5 μM), VX-509 (10 μM), or combinations for 10 days. The media were replenished every three days. The 3D cultures’ size was measured on day ten by ImageJ. The organoid tumour area in the vehicle-treated group was normalised as 100%. Scale bar, 50 μm. *N* = 6–12 replicates. Data are mean ± s.e.m. and were analysed with Brown-Forsythe and Welch ANOVA (**b**, **c**, **d**). *p < 0.05; **p < 0.01; ****p < 0.0001.

Our in vitro and in vivo data have demonstrated that AICAR inhibits lung tumour cells’ growth and survival by degrading MUC1 and dampening interactions of MUC1-JAK1 and MUC1-EGFR (Fig. 6a). We asked if blocking EGFR and JAK signalling would increase treatment efficacy of AICAR. To answer this question, we co-treated H1975 cells with VX-509, osimertinib, and AICAR for three days and examined their cell viability. Our data demonstrated that AICAR treatment alone is robust in inhibiting tumour cells’ viability compared with VX-509 and osimertinib. Therapy in a combination of the three molecules showed the most cell growth inhibition (*p* < 0.05) (Fig. 6b). Next, we established 3D organoids from one *EGFR*-mutant PDX tumour (Table S9). Treatment with AICAR for 10 days decreased the growth of tumour organoids (*p* < 0.05). Co-treating organoids with AICAR, VX-509, and osimertinib further reduced organoid growth significantly (*p* < 0.01) (Fig. 6c). Consistently, co-treating *EGFR* transgenic mouse lung tumour tissue-derived organoids with osimertinib, VX-509, and AICAR significantly inhibited tumour organoid initiation in 10 days (*p* < 0.05) (Fig. 6d). These data suggest that blocking MUC1, JAK, and EGFR using AICAR, VX-509, and osimertinib can reduce tumour cells’ survival and growth.

## Discussion

MUC1 is a druggable target in the anticancer therapy [99]. Even though peptide- and siRNA-based approaches have the potential to target MUC1, small molecule therapeutics have many advantages due to their cell-permeable and potent features in the clinical treatment [24, 100]. Currently, no small molecules were reported to target oncogenic MUC1 directly. Even though small molecule apigenin was reported to inhibit MUC1-CT dimerisation in the breast cancer cell lines, the inhibitory effect was probably mediated by blocking other direct targets such as heterogeneous nuclear ribonucleoprotein A2 and the oncogenic signalling pathways [34–36]. Our study was the first to describe the intrinsic metabolite AICAR physically binding and targeting MUC1 to induce lung tumour cell apoptosis. Our western blot data showed that MUC1-CT expression was decreased by both time- and dose-dependent treatments of AICAR. This was consistent with the data from the in silico and thermal stability assays that AICAR directly binds to MUC1. It is unclear why AICAR’s binding to MUC1-CT reduces MUC1-CT protein stability. A previous study showed that peroxisome proliferator-activated receptor gamma (PPARγ) E3 ubiquitin ligase induced MUC1-CT ubiquitination and decreased MUC1-CT oncoprotein stability [101]. It is unlikely that PPARγ degrades MUC1-CT directly because the in silico analysis does not support PPARγ as the direct binding target of AICAR. Our RNA-seq data found no significant changes in PPARγ expression after AICAR treatment. Thus, AICAR-induced MUC1 instability might be PPARγ-independent. It will be interesting to explore if AICAR-induced MUC1-CT instability is correlated to MUC1-CT ubiquitination in the future.

Recent research has shown that the tumour microenvironment has played a vital role in promoting tumour initiation and progression by modulating the extracellular matrix and immune cell homing [102]. We noticed that AICAR treatment could block the proliferation of stromal cells, including alveolar macrophages, endothelial cells, and fibroblasts. Tumour-adjacent stromal cells promote tumour initiation and progression by providing paracrine signals [103]. Thus, AICAR might concurrently decrease tumour cells’ survival by inhibiting paracrine signalling from these tumour stromal cells. Recent studies showed that blocking JAK-STAT signalling with the JAK inhibitors reduced tumour-promoting inflammation and tumour formation in the lungs [56]. Our data showing reduced JAK-STAT signalling after AICAR treatment indicates that AICAR might play a similar role to JAK inhibitors in blocking pro-tumorigenic signals from stromal cells. A non-specific toxicity concern might arise due to targeting non-tumour cells. In the lung tumour mouse xenograft model, we did not observe significant body weight loss and liver phenotypic changes in the mice during the treatment with AICAR, supporting the safety of the application of AICAR in treating the tumour in the preclinical mouse models.

Besides AICAR targeting MUC1, our data suggest that AICAR also blocks JAK1 phosphorylation. A previous study showed that MUC1 interacts with STAT3 directly in breast cancer [30]. It is not clear if MUC1 interacts with JAK1 directly in lung cancer. Our study is the first to report that AICAR treatment impairs MUC1 and JAK1 interaction. Besides JAK1, mutant *EGFR* mediates MUC1-CT expression in the transgenic lung cancer mouse model. This is consistent with a previous study in breast cancer demonstrating that p-EGFR activates MUC1 by phosphorylating MUC1 [29]. Our in vivo model confirms the regulation of MUC1 by mutant *EGFR*. In turn, MUC1-CT could also control the JAK and EGFR signalling pathways. This indicates that JAK1/EGFR-MUC1 might form a positive feedback loop to promote tumour cell proliferation and survival.

Even though previous studies support AICAR’s treatment in leukaemia, hepatocarcinoma, and prostate cancer, our cell-based screening of cytotoxicity of AICAR was limited to its relatively smaller scale in lung cancer. Our data calls for high-throughput screening of various cancer cell lines in combination treatment with AICAR, JAK, or EGFR inhibitors. Intratumoral and intertumoral heterogeneity in MUC1 expression across diverse types of lung cancer will challenge the strategy of applying AICAR in targeting MUC1-expressing cells. Our study warrants lung cancer patients’ stratification in future clinical trials. Our study was also limited to MUC1–JAK1 interaction in the same type of lung cancer cells. We could not exclude the binding of JAK1 in one kind of cell to MUC1 expressed in another type of cell. It will be interesting to explore whether AICAR treatment can concurrently target tumour and tumour-adjacent cells by blocking the protein–protein interactions. Despite these limitations, our discovery in AICAR paves a new way to block lung tumour growth by blocking MUC1 and its interacting proteins including JAK1 and EGFR. Thus, we have found a new compound to block MUC1-CT in lung cancer cells that might apply to many other types of cancers.

## Supplementary information

Supplemental information

## Data Availability

Data from this study have been deposited in the Gene Expression Omnibus (GEO) databases under the following accession: GSE198777 (RNA seq). The results shown in this manuscript were partially based upon data generated by the Lung Cancer Explorer portal (https://lce.biohpc.swmed.edu/lungcancer/) and the Genentech dataset (EGAD00001000725). The genetic mutation status was confirmed by the cansar portal (v3.0 beta) (https://cansar.icr.ac.uk/) and the cancer Catalogue Of Somatic Mutations In Cancer (COSMIC) (http://cancer.sanger.ac.uk/cosmic/sample/overview?id=722040). The protein names were validated by Protein Knowledgebase (UniProtKB) (version: February 2, 2022) (https://www.uniprot.org/uniprot/). The file checksum was generated using MD5 File Checksum (http://emn178.github.io/online-tools/md5_checksum.html) to deposit RNA-seq data to Gene Expression Omnibus (GEO). The results shown in this manuscript were partly based upon data generated by the TCGA Research Network: http://cancergenome.nih.gov/. The data that support the plots within this paper are available from the corresponding author upon reasonable request.
