# Supplementary material for: An intrinsic purine metabolite AICAR blocks lung tumour growth by targeting oncoprotein mucin 1
Source: Br J Cancer. 2023 Feb 21;128(9):1647–64. doi: 10.1038/s41416-023-02196-z (PMC10133251; doi:10.1038/s41416-023-02196-z)
Supplement: Supplementary file 1 — Supplemental information [file 41416_2023_2196_MOESM1_ESM.docx]

Supplementary Data for

**An intrinsic purine metabolite AICAR blocks lung tumor growth by targeting oncoprotein mucin 1**

Fareesa Aftab,^1*^ Alice Rodriguez-Fuguet,^1*^ Luis Silva,^1*^ Ikei S. Kobayashi,^2^ Jiao Sun,^3^ Katerina Politi,^4^ Elena Levantini,^5, 6^ Wei Zhang,^3^ Susumu S. Kobayashi, ^2, 7^ Wen Cai Zhang^1†^

^†^Corresponding author. Email: wencai.zhang[@ucf.edu](mailto:xxxxx@xxxx.xxx)

**This PDF file includes:**

Figs. S1 to S9

Tables S1 to S9


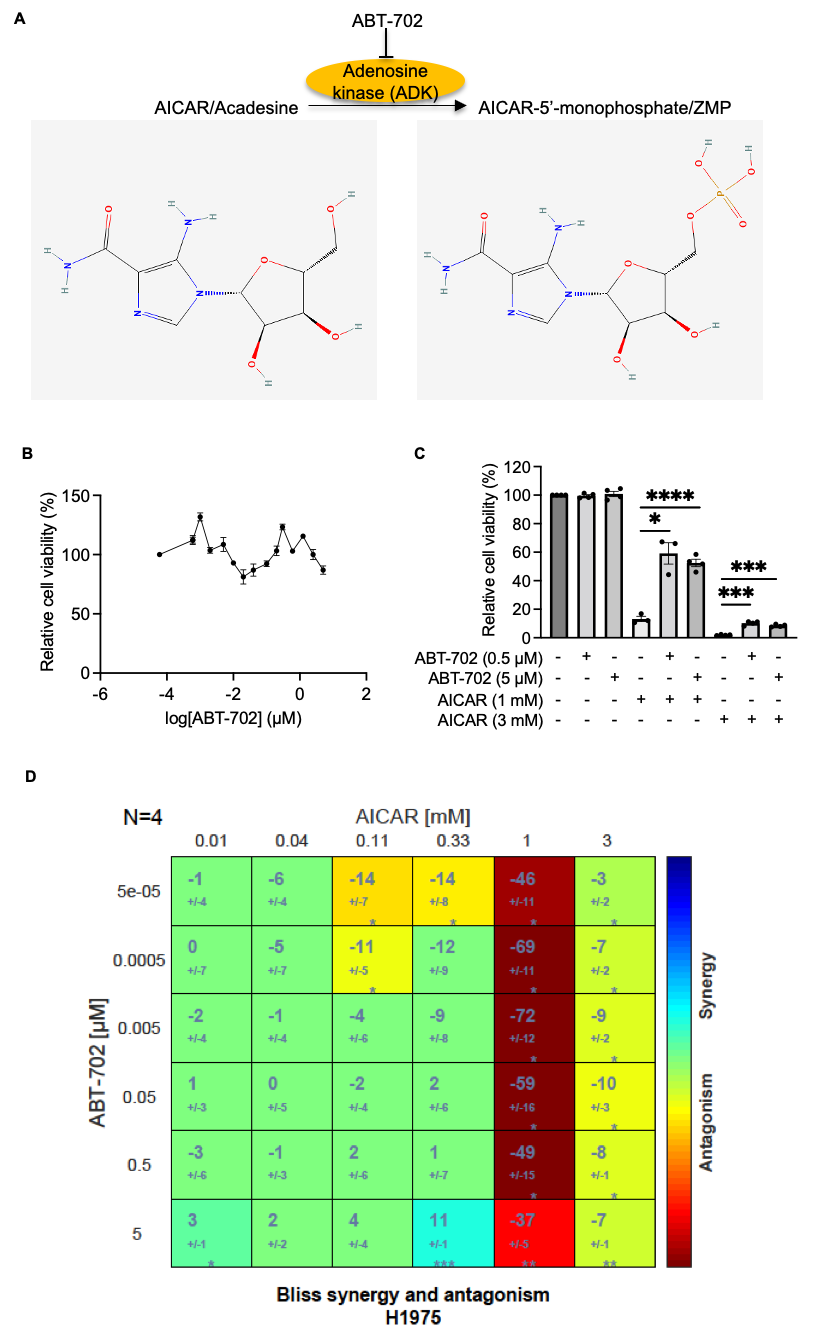


Fig. S1.

**Small molecule ABT-702 inhibits adenosine kinase and blocks conversion from AICAR to ZMP.**

1. The reaction of AICAR conversion to ZMP catalyzed by adenosine kinase (ADK). ABT-702 inhibits ADK and blocks ADK-catalyzed conversion. The chemical structures of AICAR and ZMP are shown below.
2. Cell viability assay for ABT-702 in H1975 cells. 3,000 cells were plated and treated with serially diluted ABT-702 (0~10 μM). Cell viability was measured with CellTiter-Glo assay three days after treatment. N= 3 replicates.
3. Cell viability of H1975 cells treated with a vehicle, AICAR (1 and 3 mM), and ABT-702 (0.5 and 5 μM) for three days. N= 4 replicates.
4. A synergistic assay using AICAR (0~3 mM) and ABT-702 (0~5 μM) in H1975 cells. 3,000 cells were plated and treated with serially diluted AICAR and ABT-702. Cell viability was measured with CellTiter-Glo assay three days after treatment. N= 4 replicates.

Data are mean ± s.e.m. and were analyzed with one-way ANOVA (C, D). *, *p*<0.05; **, *p*<0.01; ***, *p*<0.001; ****, *p*<0.0001.

Fig. S2.

**Top AICAR and ZMP-binding proteins screened by FINDSITE^comb2.0^.**

A Venn diagram showing the top AICAR/acadesine-specific binding targets (left panel), ZMP (AICAR-5’-monophosphate) binding targeting (right panel), and overlapping targets between AICAR and ZMP (middle panel).


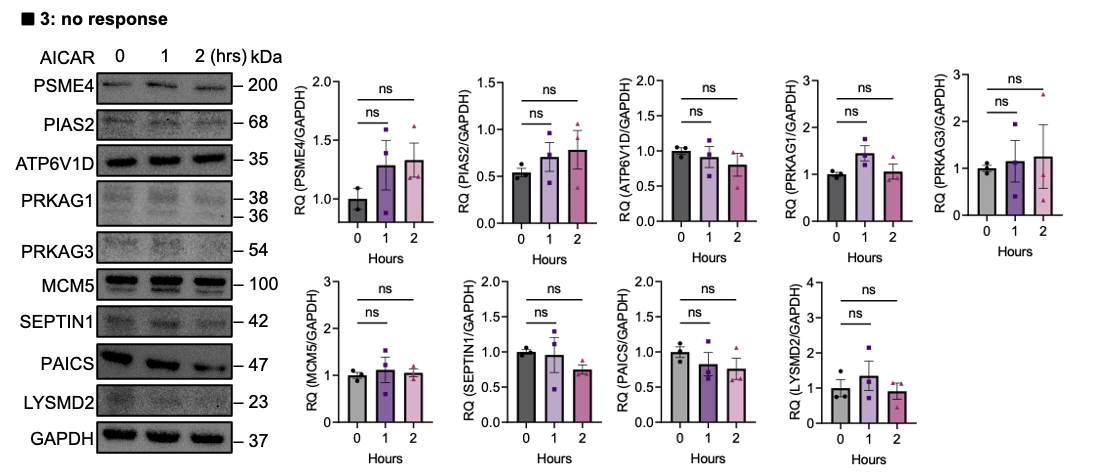


Fig. S3.

**AICAR treatment does not change the expression levels of other targets in lung cancer cells.**

Time-dependent western blotting and relative quantification of protein expression for AICAR binding proteins (type 3). No treatment responses on H1975 cells treated with 1 mM AICAR for one and two hours were demonstrated. GAPDH was used as a loading control. N=2~3 replicates. ns, not significant.


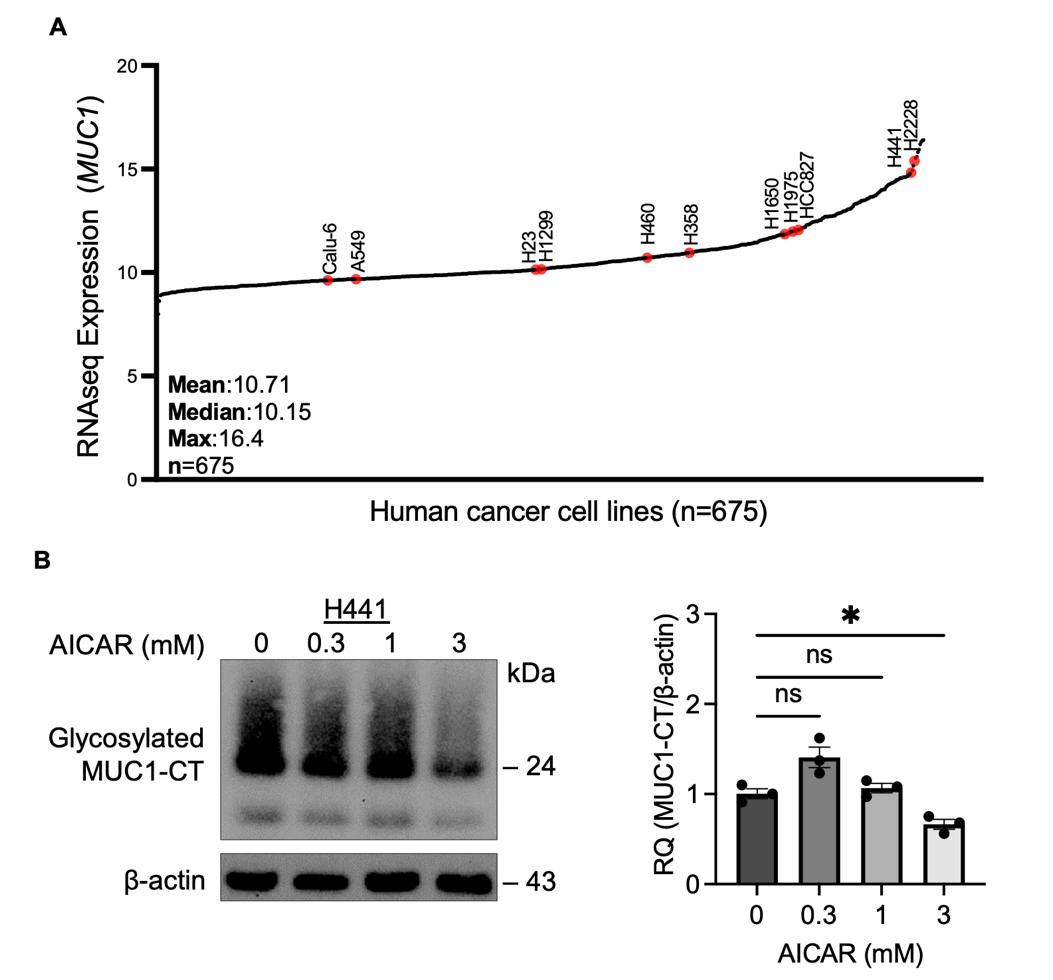


Fig. S4.

**AICAR inhibits MUC1-CT expression in lung cancer cell lines.**

1. *MUC1* gene expression in 675 human cancer cell lines from the Genentech dataset.
2. Western blot assay and relative quantification of dose-dependent expression for MUC1-CT in H441 cell line. The cells were treated with increasing doses of AICAR (0, 0.3, 1, and 3 mM) for four hours, followed by protein extraction and western blot assay. β-actin was used as a loading control. N=3 replicates.

Data are mean ± s.e.m. and were analyzed with Brown-Forsythe and Welch one-way ANOVA (B). *, *p*<0.05; ns, not significant.


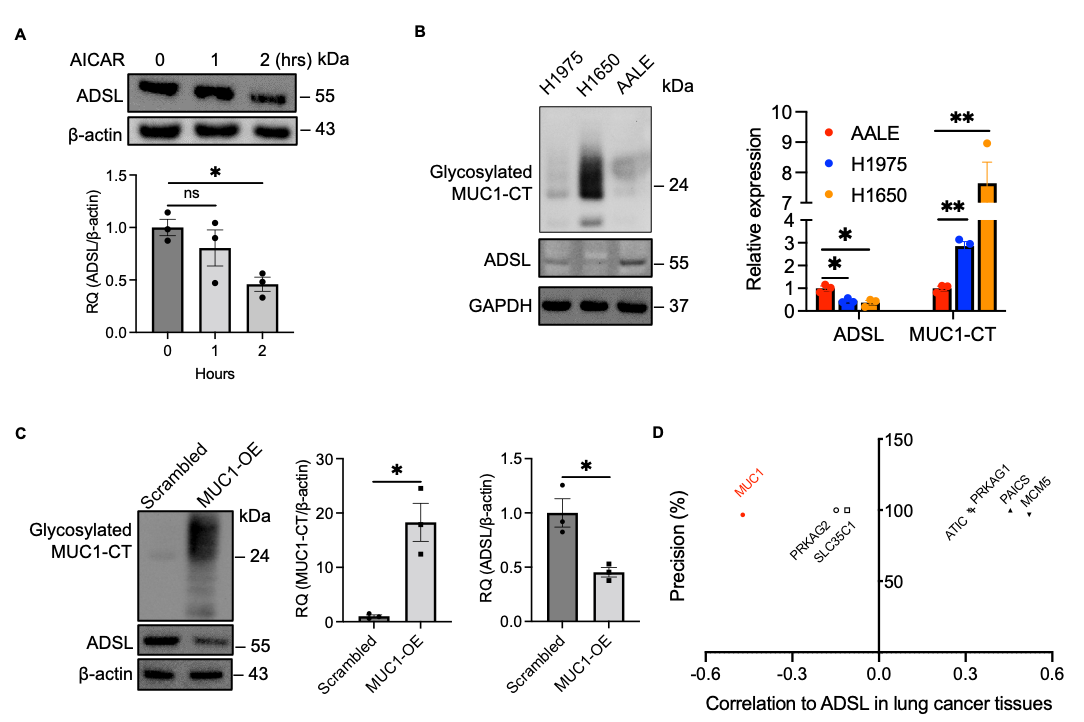


Fig. S5.

MUC1 is negatively correlated to the expression level of ADSL.

1. Longitudinal analysis of ADSL expression by western blotting and relative quantification in H1975 cells treated with AICAR. The cells were treated with 1 mM AICAR for one and two hours, followed by a western blot assay. β-actin was used as a loading control. N=3 replicates.
2. Western blotting and relative quantification of expression for MUC1-CT and ADSL across three human lung cell lines (H1975, H1650, AALE). The relative protein expression levels in AALE cells were normalized as 1. GAPDH was used as a loading control. N=3 replicates.
3. Western blotting and relative expression quantification for MUC1-CT and ADSL expression in H1975 cells with MUC1 overexpression (OE). β-actin was used as a loading control. N=3 replicates.
4. Analysis of top candidates correlated to *ADSL* gene expression levels in human lung cancer tissues from six datasets. The precision (%) score analyzed by the FINDSITE^comb2.0^ at 95% was used as a cut-off. N=1,418.

Data are mean ± s.e.m. and were analyzed with Brown-Forsythe and Welch one-way ANOVA (A); unpaired two-tailed *t*-test (B, C); Pearson correlation analysis (D). *, *p*<0.05; **, *p*<0.01; ns, not significant.

Fig. S6.

**A diagram of the KEGG signaling pathway showing enriched genes in the JAK-STAT signaling pathway in H1975 cells treated with AICAR compared with vehicle-treated cells.** GF, growth factor.


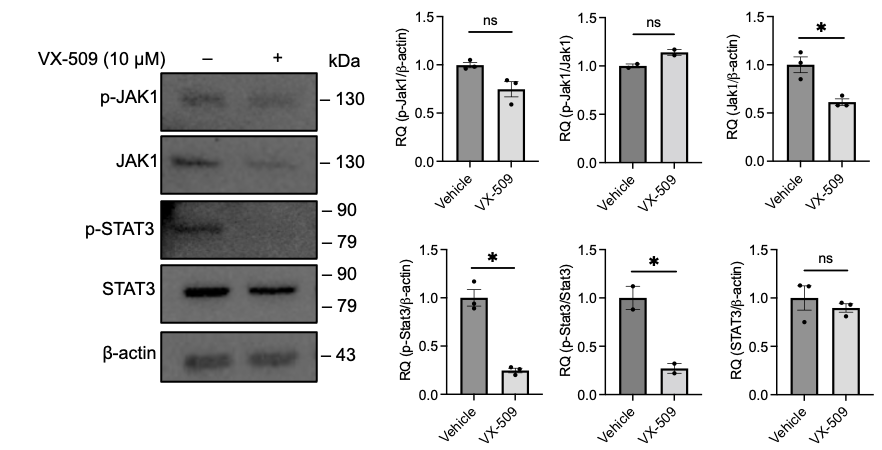


Fig. S7.

**Western blot assay and relative expression quantification for JAK1 signaling in H1975 cells treated with VX-509.** The cells were treated with a pan-JAK inhibitor VX-509 (10 μM) for four hours, followed by a western blot assay for p-JAK1, JAK1, p-STAT3, and STAT3. β-actin was used as a loading control. N=3 replicates.

Data are mean ± s.e.m. and were analyzed with Welch’s t-test. *, *p*<0.05; ns, not significant.

Fig. S8.

**Patients’ overall and disease-free survival in lung adenocarcinoma patients at stage I.**

1. Patients’ overall survival in lung adenocarcinoma patients at stage I. The median expression levels of *MUC1* were used for a cut-off of high and low expression for *MUC1*. N=272.
2. Patients’ disease-free survival in lung adenocarcinoma patients at stage I. The median expression levels of *MUC1* were used for a cut-off of high and low expression for *MUC1*. N=49.

Data were analyzed with a log-rank test (A, B).


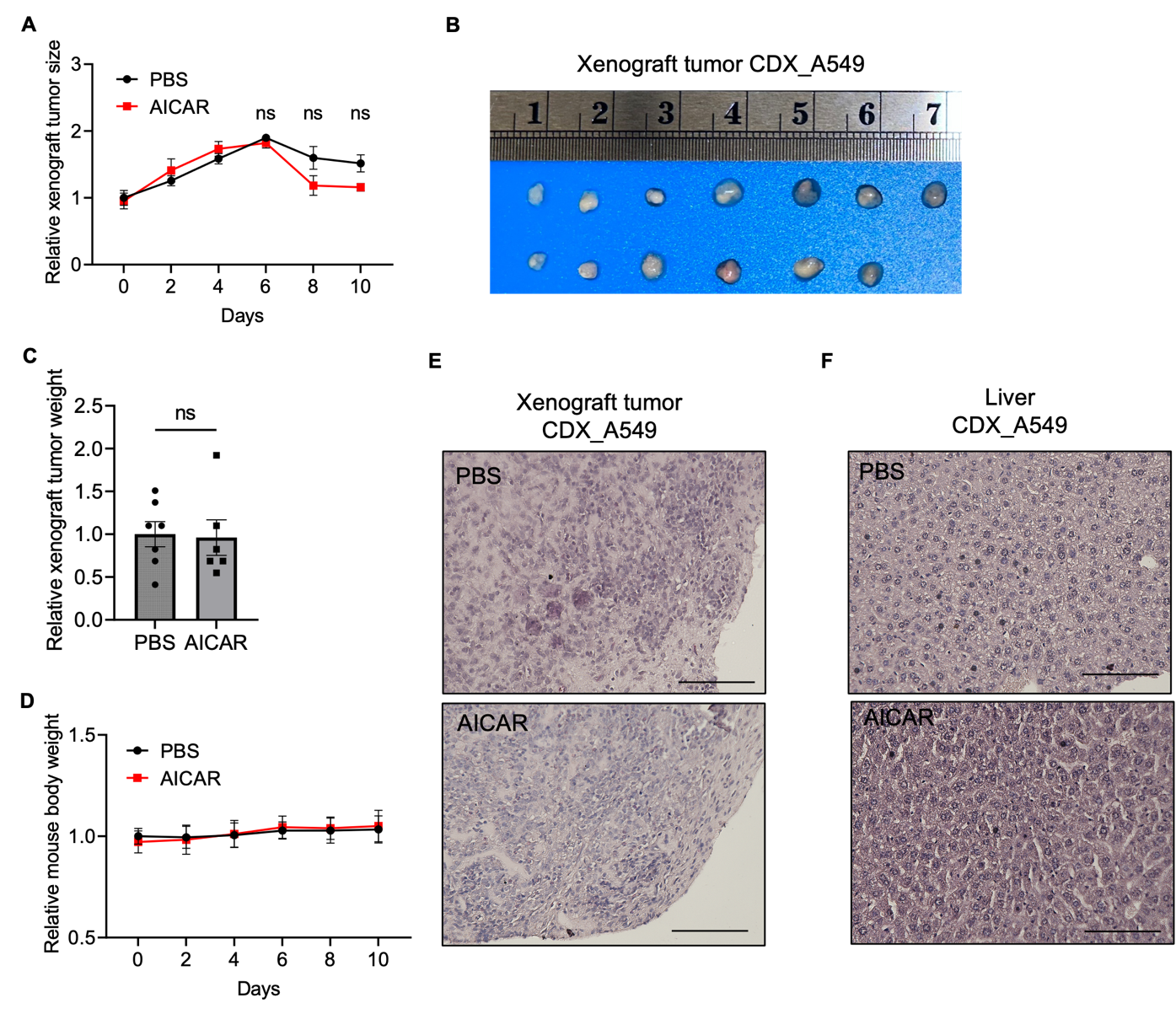


Fig. S9.

**AICAR does not block A549-derived xenograft tumor formation in mice.**

1. Xenograft tumor growth in mice treated with AICAR. The xenograft tumor was pre-established by implanting 1 million A549 cells subcutaneously. When the tumor reached 45 mm^3^, the mice were treated with 300 mg/kg/day AICAR in PBS or a vehicle subcutaneously for ten days. The tumor was measured with a digital caliper, and the tumor size was calculated. N= 6~7 replicates.

**(B,C)** Xenograft tumor images (B) and relative weight quantification (C) from mice treated with AICAR or PBS. The average tumor weight from the PBS-treated group is normalized as 1. N= 6~7 replicates.

**(D)** Mouse body weight after treatment with AICAR or PBS for ten days. N= 6~7 replicates.

**(E,F)** H&E staining of subcutaneous tumors (E) and liver tissues (F) from A549 cell line-derived xenograft (CDX) treated with PBS or AICAR. Scale bar, 125 μm.

Data are mean ± s.e.m. and were analyzed with Welch’s *t*-test (A, C, D). ns, not significant.

Table S1.

EC50 of AICAR in a panel of lung cell lines.

| **Category** | **Cell lines** | **EC50 (mM)** |
| --- | --- | --- |
| LUAD *EGFR* Mutant | H1975 | 0.298 |
|  | PC9 | 0.300 |
|  | H3255 | 0.320 |
|  | PC9-ER | 0.410 |
|  | H1650 | 0.420 |
|  | HCC827 | 0.880 |
| *EGFR* wild type | H441 | 0.640 |
|  | H358 | 1.280 |
|  | H69 | 1.440 |
|  | A549 | 1.600 |
|  | H23 | 1.610 |
| Non-malignant  stromal | CCD-13Lu | 1.030 |
|  | NR8383 | 1.110 |
|  | HULEC-5A | 1.450 |

Note: LUAD, lung adenocarcinoma.

Table S2.

Top 101 AICAR monophosphate-binding proteins virtually screened by FINDSITE^comb2.0^.

| Protein name | Description | Precision | mTC |
| --- | --- | --- | --- |
| SLC35C1 | GDP-fucose transporter 1 isoform a | 0.999998 | 0.974013 |
| PAICS | multifunctional protein ADE2 isoform 1 | 0.999998 | 0.963485 |
| FXYD3 | FXYD domain-containing ion transport regulator 3 isoform 3 | 0.999998 | 0.958458 |
| ATIC | bifunctional purine biosynthesis protein PURH | 0.999998 | 0.95398 |
| LYSMD2 | lysM and putative peptidoglycan-binding domain-containing protein 2 isoform 1 | 0.999998 | 0.951265 |
| PRKAG2 | 5'-AMP-activated protein kinase subunit gamma-2 isoform c | 0.999998 | 0.95122 |
| PRKAG3 | 5'-AMP-activated protein kinase subunit gamma-3 | 0.999998 | 0.95116 |
| PRKAG1 | 5'-AMP-activated protein kinase subunit gamma-1 isoform 1 | 0.999998 | 0.950864 |
| ATP6V1D | V-type proton ATPase subunit D | 0.967031 | 0.946028 |
| RBFA | putative ribosome-binding factor A, mitochondrial isoform 1 precursor | 0.967031 | 0.943889 |
| MCM5 | DNA replication licensing factor MCM5 | 0.967031 | 0.943544 |
| MUC1 | mucin-1 isoform 3 precursor | 0.967031 | 0.935843 |
| IKZF5 | zinc finger protein Pegasus | 0.967031 | 0.932018 |
| PSME4 | proteasome activator complex subunit 4 | 0.967031 | 0.929994 |
| TMEM70 | transmembrane protein 70, mitochondrial isoform a | 0.967031 | 0.928655 |
| PCDHA5 | protocadherin alpha-5 isoform 1 precursor | 0.967031 | 0.924528 |
| MYO1D | myosin-Id | 0.829664 | 0.700397 |
| SEPTIN1 | septin-1 | 0.827833 | 0.700397 |
| TP63 | tumor protein 63 isoform 5 | 0.827371 | 0.700397 |
| TP73 | tumor protein p73 isoform d | 0.82733 | 0.700397 |
| TH | tyrosine 3-monooxygenase isoform b | 0.826367 | 0.700397 |
| CCNY | cyclin-Y isoform 2 | 0.825754 | 0.700397 |
| RDM1 | RAD52 motif-containing protein 1 isoform 2 | 0.825344 | 0.700397 |
| MRPS11 | 28S ribosomal protein S11, mitochondrial isoform a | 0.829664 | 0.700397 |
| PIAS2 | E3 SUMO-protein ligase PIAS2 isoform alpha | 0.41336 | 0.794582 |
| NPRL2 | nitrogen permease regulator 2-like protein | 0.41336 | 0.793185 |
| MARCO | macrophage receptor MARCO | 0.41336 | 0.791166 |
| TEX35 | Testis-expressed protein 35 | 0.41336 | 0.787908 |
| ZNF692 | zinc finger protein 692 isoform 3 | 0.41336 | 0.787555 |
| CLASRP | CLK4-associating serine/arginine rich protein | 0.41336 | 0.78653 |
| FCF1 | rRNA-processing protein FCF1 homolog | 0.41336 | 0.780803 |
| CBFB | core-binding factor subunit beta isoform 2 | 0.41336 | 0.777537 |
| PRKAB2 | 5'-AMP-activated protein kinase subunit beta-2 | 0.41336 | 0.762182 |
| ACSS2 | acetyl-coenzyme A synthetase, cytoplasmic isoform 2 | 0.41336 | 0.759581 |
| ACSS1 | acetyl-coenzyme A synthetase 2-like, mitochondrial precursor | 0.41336 | 0.759581 |
| ERP29 | endoplasmic reticulum resident protein 29 isoform 2 precursor | 0.41336 | 0.759581 |
| ACSS3 | acyl-CoA synthetase short-chain family member 3, mitochondrial precursor | 0.41336 | 0.759581 |
| ACSL5 | long-chain-fatty-acid--CoA ligase 5 isoform b | 0.41336 | 0.759581 |
| ADCY1 | adenylate cyclase type 1 | 0.41336 | 0.759581 |
| ACSL6 | long-chain-fatty-acid--CoA ligase 6 isoform b | 0.41336 | 0.759581 |
| ACSL1 | long-chain-fatty-acid--CoA ligase 1 | 0.41336 | 0.759581 |
| NOPCHAP1 | NOP protein chaperone 1 | 0.41336 | 0.759543 |
| FAM76A | Protein FAM76A | 0.41336 | 0.758322 |
| FDXACB1 | ferredoxin-fold anticodon-binding domain-containing protein 1 | 0.41336 | 0.75782 |
| STEEP1 | STING ER exit protein | 0.41336 | 0.757592 |
| MAGEB18 | melanoma-associated antigen B18 | 0.41336 | 0.757592 |
| BTG3 | protein BTG3 isoform a | 0.41336 | 0.757592 |
| ASPDH | putative L-aspartate dehydrogenase isoform 2 | 0.41336 | 0.757592 |
| TMEM143 | transmembrane protein 143 | 0.41336 | 0.757592 |
| ISYNA1 | inositol-3-phosphate synthase 1 isoform 3 | 0.41336 | 0.757592 |
| RFLNB | Refilin-B | 0.41336 | 0.757592 |
| CNOT1 | CCR4-NOT transcription complex subunit 1 isoform b | 0.41336 | 0.757592 |
| GALNT9 | polypeptide N-acetylgalactosaminyltransferase 9 isoform A | 0.41336 | 0.757592 |
| PFKM | 6-phosphofructokinase, muscle type isoform 1 | 0.41336 | 0.757592 |
| PHRF1 | PHD and RING finger domain-containing protein 1 | 0.41336 | 0.757592 |
| TTC16 | tetratricopeptide repeat protein 16 | 0.41336 | 0.757592 |
| XPA | DNA repair protein complementing XP-A cells | 0.41336 | 0.757592 |
| FBXO10 | F-box only protein 10 | 0.41336 | 0.757592 |
| POLM | DNA polymerase mu | 0.41336 | 0.757592 |
| METTL24 | Probable methyltransferase-like protein 24 | 0.41336 | 0.757592 |
| UBR2 | E3 ubiquitin-protein ligase UBR2 isoform 1 | 0.41336 | 0.757592 |
| SLC30A5 | zinc transporter 5 isoform 2 | 0.41336 | 0.757592 |
| NADK2 | NAD kinase 2, mitochondrial | 0.41336 | 0.757592 |
| ROPN1L | ropporin-1-like protein | 0.41336 | 0.757592 |
| VEPH1 | ventricular zone-expressed PH domain-containing protein homolog 1 isoform 5 | 0.41336 | 0.757592 |
| HOXD11 | homeobox protein Hox-D11 | 0.41336 | 0.757592 |
| OTX1 | homeobox protein OTX1 | 0.41336 | 0.757592 |
| IRF2BP2 | interferon regulatory factor 2-binding protein 2 isoform A | 0.41336 | 0.757592 |
| AHCYL1 | S-adenosylhomocysteine hydrolase-like protein 1 | 0.41336 | 0.757592 |
| PLPPR5 | lipid phosphate phosphatase-related protein type 5 isoform 1 | 0.41336 | 0.757592 |
| LDLRAP1 | low density lipoprotein receptor adapter protein 1 | 0.41336 | 0.757592 |
| NADK | NAD kinase | 0.41336 | 0.757592 |
| TFAP2A | transcription factor AP-2-alpha isoform c | 0.41336 | 0.757558 |
| CNNM2 | metal transporter CNNM2 isoform 2 | 0.41336 | 0.757003 |
| UBA3 | NEDD8-activating enzyme E1 catalytic subunit isoform 1 | 0.41336 | 0.756929 |
| SYN1 | synapsin-1 isoform Ib | 0.41336 | 0.756648 |
| NMNAT1 | nicotinamide mononucleotide adenylyltransferase 1 | 0.41336 | 0.756612 |
| SYN3 | synapsin-3 isoform IIIg | 0.41336 | 0.756558 |
| ATG7 | ubiquitin-like modifier-activating enzyme ATG7 isoform c | 0.41336 | 0.756545 |
| SYN2 | synapsin-2 isoform Iia | 0.41336 | 0.756319 |
| NDST2 | bifunctional heparan sulfate N-deacetylase/N-sulfotransferase 2 | 0.41336 | 0.756145 |
| PLA2G12B | group XIIB secretory phospholipase A2-like protein precursor | 0.41336 | 0.756145 |
| RLN1 | prorelaxin H1 preproprotein | 0.41336 | 0.756145 |
| NDST1 | bifunctional heparan sulfate N-deacetylase/N-sulfotransferase 1 | 0.41336 | 0.756145 |
| NDST3 | bifunctional heparan sulfate N-deacetylase/N-sulfotransferase 3 | 0.41336 | 0.756145 |
| NDST4 | bifunctional heparan sulfate N-deacetylase/N-sulfotransferase 4 | 0.41336 | 0.756145 |
| SULT1B1 | sulfotransferase family cytosolic 1B member 1 | 0.41336 | 0.756145 |
| SULT1C4 | sulfotransferase 1C4 | 0.41336 | 0.756145 |
| SULT1C3 | sulfotransferase 1C3 | 0.41336 | 0.756145 |
| BSDC1 | BSD domain-containing protein 1 isoform d | 0.41336 | 0.75608 |
| RIMKLA | ribosomal protein S6 modification-like protein A | 0.41336 | 0.75563 |
| RNASE8 | ribonuclease 8 precursor | 0.41336 | 0.754879 |
| RNASE7 | ribonuclease 7 precursor | 0.41336 | 0.754879 |
| RNASE2 | non-secretory ribonuclease precursor | 0.41336 | 0.754879 |
| AK9 | adenylate kinase domain-containing protein 1 isoform 1 | 0.41336 | 0.754862 |
| SWSAP1 | ATPase SWSAP1 | 0.41336 | 0.75457 |
| TIAL1 | nucleolysin TIAR isoform 2 | 0.41336 | 0.75413 |
| NMNAT3 | nicotinamide mononucleotide adenylyltransferase 3 | 0.41336 | 0.75413 |
| NMNAT2 | nicotinamide mononucleotide adenylyltransferase 2 isoform 1 | 0.41336 | 0.75413 |
| CMPK1 | UMP-CMP kinase isoform a | 0.41336 | 0.754062 |
| DAP3 | 28S ribosomal protein S29, mitochondrial | 0.41336 | 0.753947 |

Note: mTC, modified Tanimoto coefficient.

Table S3.

Top 101 AICAR/acadesine-binding proteins virtually screened by FINDSITE^comb2.0^.

| Protein name | Description | Precision | mTC |
| --- | --- | --- | --- |
| TMEM70 | transmembrane protein 70, mitochondrial isoform a | 0.967031 | 0.926952 |
| MYO1D | Myosin1D protein | 0.967031 | 0.917556 |
| SEPTIN1 | septin-1 | 0.967031 | 0.91593 |
| TP63 | tumor protein 63 isoform 4 | 0.967031 | 0.915173 |
| TP73 | tumor protein p73 isoform d | 0.967031 | 0.915166 |
| TH | tyrosine 3-monooxygenase isoform b | 0.967031 | 0.914619 |
| CCNY | cyclin-Y isoform 2 | 0.967031 | 0.914296 |
| RDM1 | RAD52 motif-containing protein 1 isoform 2 | 0.967031 | 0.913599 |
| SLC35C1 | GDP-fucose transporter 1 isoform a | 0.88404 | 0.873316 |
| PAICS | multifunctional protein ADE2 isoform 1 | 0.88404 | 0.862267 |
| FXYD3 | FXYD domain-containing ion transport regulator 3 isoform 3 | 0.88404 | 0.86091 |
| ATIC | bifunctional purine biosynthesis protein PURH | 0.88404 | 0.857196 |
| LYSMD2 | lysM and putative peptidoglycan-binding domain-containing protein 2 isoform 1 | 0.88404 | 0.855156 |
| PRKAG2 | 5'-AMP-activated protein kinase subunit gamma-2 isoform c | 0.88404 | 0.85343 |
| ATP6V1D | V-type proton ATPase subunit D | 0.700397 | 0.848808 |
| MCM5 | DNA replication licensing factor MCM5 | 0.700397 | 0.848459 |
| RBFA | putative ribosome-binding factor A, mitochondrial isoform 1 precursor | 0.700397 | 0.846912 |
| MUC1 | mucin-1 isoform 3 precursor | 0.700397 | 0.842523 |
| PSME4 | proteasome activator complex subunit 4 | 0.700397 | 0.841094 |
| PCDHA5 | protocadherin alpha-5 isoform 1 precursor | 0.700397 | 0.839054 |
| TBXAS1 | thromboxane-A synthase 1 isoform 4 | 0.41336 | 0.793745 |
| CYP39A1 | 24-hydroxycholesterol 7-alpha-hydroxylase | 0.41336 | 0.793697 |
| CYP3A4 | cytochrome P450 3A4 | 0.41336 | 0.793656 |
| OR2Z1 | olfactory receptor 2Z1 | 0.41336 | 0.793653 |
| CYP3A43 | cytochrome P450 3A43 isoform 2 | 0.41336 | 0.793646 |
| CYP3A7 | cytochrome P450 3A7 | 0.41336 | 0.793646 |
| CYP46A1 | cholesterol 24-hydroxylase | 0.41336 | 0.793615 |
| CYP4Z1 | cytochrome P450 4Z1 | 0.41336 | 0.793615 |
| CYP4X1 | cytochrome P450 4X1 | 0.41336 | 0.793615 |
| CHRM5 | muscarinic acetylcholine receptor M5 | 0.41336 | 0.79361 |
| CHRM1 | muscarinic acetylcholine receptor M1 | 0.41336 | 0.79361 |
| CHRM3 | muscarinic acetylcholine receptor M3 | 0.41336 | 0.79361 |
| CYP3A5 | cytochrome P450 3A5 isoform 1 | 0.41336 | 0.793593 |
| CYP20A1 | cytochrome P450 20A1 | 0.41336 | 0.793551 |
| CYP26B1 | cytochrome P450 26B1 | 0.41336 | 0.793542 |
| POLDIP3 | polymerase delta-interacting protein 3 isoform 2 | 0.41336 | 0.79354 |
| RFESD | Rieske domain-containing protein isoform 1 | 0.41336 | 0.793536 |
| SYNPO | synaptopodin isoform A | 0.41336 | 0.793525 |
| TWIST1 | twist-related protein 1 | 0.41336 | 0.793514 |
| TMED3 | transmembrane emp24 domain-containing protein 3 precursor | 0.41336 | 0.793506 |
| MAN2A2 | alpha-mannosidase 2x | 0.41336 | 0.793483 |
| CYP7B1 | 25-hydroxycholesterol 7-alpha-hydroxylase | 0.41336 | 0.793474 |
| PTGIS | prostacyclin synthase | 0.41336 | 0.793461 |
| CYP2D6 | cytochrome P450 2D6 isoform 2 | 0.41336 | 0.793459 |
| RSAD2 | radical S-adenosyl methionine domain-containing protein 2 | 0.41336 | 0.793455 |
| CYP4F11 | cytochrome P450 4F11 | 0.41336 | 0.793443 |
| CYP4F2 | Cytochrome P450 4F2 | 0.41336 | 0.793443 |
| CYP4F12 | cytochrome P450 4F12 | 0.41336 | 0.793443 |
| CYP4F3 | leukotriene-B(4) omega-hydroxylase 2 | 0.41336 | 0.793443 |
| CYP4F8 | cytochrome P450 4F8 | 0.41336 | 0.793443 |
| CYP4F22 | cytochrome P450 4F22 | 0.41336 | 0.793443 |
| CYP4V2 | cytochrome P450 4V2 | 0.41336 | 0.793443 |
| CYP4A22 | cytochrome P450 4A22 precursor | 0.41336 | 0.793443 |
| CYP4A11 | cytochrome P450 4A11 | 0.41336 | 0.793443 |
| CYP4B1 | cytochrome P450 4B1 isoform a | 0.41336 | 0.793443 |
| CYP2W1 | cytochrome P450 2W1 | 0.41336 | 0.793437 |
| PATJ | inaD-like protein | 0.41336 | 0.793435 |
| RHBDD2 | rhomboid domain-containing protein 2 isoform b | 0.41336 | 0.793426 |
| CYP21A2 | steroid 21-hydroxylase isoform a | 0.41336 | 0.793419 |
| CYP2U1 | cytochrome P450 2U1 | 0.41336 | 0.793415 |
| CYP1B1 | cytochrome P450 1B1 | 0.41336 | 0.793407 |
| COLQ | acetylcholinesterase collagenic tail peptide isoform III precursor | 0.41336 | 0.793404 |
| CYP24A1 | 1,25-dihydroxyvitamin D(3) 24-hydroxylase, mitochondrial isoform 2 precursor | 0.41336 | 0.793402 |
| CYP11A1 | cholesterol side-chain cleavage enzyme, mitochondrial isoform b | 0.41336 | 0.793402 |
| CYP27A1 | sterol 26-hydroxylase, mitochondrial precursor | 0.41336 | 0.793402 |
| CYP2R1 | vitamin D 25-hydroxylase | 0.41336 | 0.7934 |
| POLR1E | DNA-directed RNA polymerase I subunit RPA49 | 0.41336 | 0.793397 |
| CYP2C8 | cytochrome P450 2C8 | 0.41336 | 0.793382 |
| CYP2C9 | cytochrome P450 2C9 | 0.41336 | 0.793382 |
| CYP2C18 | cytochrome P450 2C18 isoform 2 | 0.41336 | 0.793382 |
| CYP1A1 | cytochrome P450 1A1 | 0.41336 | 0.793379 |
| CYP1A2 | cytochrome P450 1A2 | 0.41336 | 0.793347 |
| CYP2F1 | cytochrome P450 2F1 | 0.41336 | 0.793338 |
| CYP2B6 | cytochrome P450 2B6 | 0.41336 | 0.793338 |
| CYP2E1 | cytochrome P450 2E1 | 0.41336 | 0.793338 |
| CYP2C19 | cytochrome P450 2C19 | 0.41336 | 0.793338 |
| CYP17A1 | steroid 17-alpha-hydroxylase/17,20 lyase | 0.41336 | 0.7933 |
| OR5C1 | olfactory receptor 5C1 | 0.41336 | 0.793263 |
| CYP2J2 | cytochrome P450 2J2 | 0.41336 | 0.793193 |
| CYP2S1 | cytochrome P450 2S1 | 0.41336 | 0.793188 |
| CYP2A13 | cytochrome P450 2A13 | 0.41336 | 0.793188 |
| CYP2A7 | cytochrome P450 2A7 isoform 1 | 0.41336 | 0.793188 |
| CYP2A6 | cytochrome P450 2A6 | 0.41336 | 0.793188 |
| CYP19A1 | cytochrome P450 19A1 | 0.41336 | 0.793142 |
| GPR162 | probable G-protein coupled receptor 162 isoform 2 | 0.41336 | 0.792911 |
| ANAPC11 | anaphase-promoting complex subunit 11 isoform 1 | 0.41336 | 0.789122 |
| FAM163A | family with sequence similarity 163, member A | 0.41336 | 0.789122 |
| TAF6L | TAF6-like RNA polymerase II p300/CBP-associated factor-associated factor 65 kDa subunit 6L | 0.41336 | 0.784987 |
| EEF2KMT | hypothetical protein LOC196483 isoform 2 | 0.41336 | 0.775987 |
| ADK | adenosine kinase isoform a | 0.41336 | 0.771273 |
| KHK | ketohexokinase isoform b | 0.41336 | 0.770598 |
| MBD5 | methyl-CpG-binding domain protein 5 | 0.41336 | 0.764147 |
| PRAMEF5 | PRAME family member 5 | 0.41336 | 0.762831 |
| PRAMEF6 | PRAME family member 6 | 0.41336 | 0.762831 |
| MRPS11 | 28S ribosomal protein S11, mitochondrial isoform a | 0.175791 | 0.72123 |
| PDRG1 | p53 and DNA damage-regulated protein 1 | 0.175791 | 0.718067 |
| CD101 | immunoglobulin superfamily member 2 precursor | 0.175791 | 0.718067 |
| NPRL2 | nitrogen permease regulator 2-like protein | 0.175791 | 0.714514 |
| CNNM2 | metal transporter CNNM2 isoform 2 | 0.175791 | 0.711273 |
| RNF144A | probable E3 ubiquitin-protein ligase RNF144A | 0.175791 | 0.709809 |
| KHDC1L | putative KHDC1-like protein | 0.175791 | 0.709629 |

Note: mTC, modified Tanimoto coefficient.

Table S4.

Correlation among top AICAR/acadesine and ZMP binding targets and *ADSL* in LCE datasets.

| Correlated to *ADSL* | Lee_2008_  GSE8894 | | Sato_2013_  GSE41271 | | TCGA_LUAD_  2016 | | Bild_2006_  GSE3141 | | Der_2014_  GSE50081 | | Botling_2013_GSE37745 | | Average R |
| --- | --- | --- | --- | --- | --- | --- | --- | --- | --- | --- | --- | --- | --- |
| Genes | R | p value | R | p value | R | p value | R | p value | R | p value | R | p value |  |
| *MUC1* | -0.52 | <0.00001 | -0.47 | <0.00001 | -0.44 | <0.00001 | -0.45 | <0.00001 | -0.53 | <0.00001 | -0.42 | <0.00001 | -0.472 |
| *PRKAG2* | -0.12 | 0.160936 | -0.21 | 0.000455 | 0.05 | 0.256442 | -0.08 | 0.403915 | -0.21 | 0.004548 | -0.32 | <0.00001 | -0.148 |
| *SLC35C1* | -0.23 | 0.006652 | -0.16 | 0.007852 | -0.1 | 0.022968 | 0.05 | 0.602265 | 0 | 1 | -0.22 | 0.001946 | -0.11 |
| *ATP6V1D* | -0.13 | 0.128581 | 0.18 | 0.007852 | 0.18 | 0.000038 | -0.19 | 0.045787 | -0.15 | 0.043854 | -0.18 | 0.001946 | -0.048 |
| *PCDHA5* | -0.28 | 0.000881 | 0.07 | 0.247292 | -0.02 | 0.650047 | -0.14 | 0.142778 | 0.12 | 0.107601 | 0.05 | 0.486455 | -0.033 |
| *LYSMD2* | -0.12 | 0.160936 | -0.01 | 0.868881 | 0.17 | 0.000103 | 0.12 | 0.209658 | -0.1 | 0.18044 | -0.13 | 0.069359 | -0.012 |
| *PRKAG3* | -0.09 | 0.293821 | 0.03 | 0.620361 | 0.04 | 0.364054 | -0.11 | 0.250435 | 0.14 | 0.060145 | 0.07 | 0.329596 | 0.013 |
| *IKZF5* | 0.08 | 0.350957 | 0.11 | 0.068551 | 0.12 | 0.006299 | 0.25 | 0.008139 | -0.31 | 0.000022 | -0.12 | 0.093873 | 0.022 |
| *FXYD3* | 0.13 | 0.128581 | 0.14 | 0.020205 | -0.08 | 0.069137 | 0.09 | 0.347534 | 0.19 | 0.010411 | 0.07 | 0.329596 | 0.09 |
| *RBFA* | 0.27 | 0.001362 | 0.11 | 0.068551 | 0.27 | <0.00001 | 0.14 | 0.142778 | 0.07 | 0.349078 | 0.11 | 0.12483 | 0.161 |
| *TMEM70* | 0.21 | 0.013431 | 0.05 | 0.408863 | 0.38 | <0.00001 | 0.33 | 0.000405 | 0.11 | 0.140449 | 0.05 | 0.486455 | 0.188 |
| *PSME4* | 0.41 | <0.00001 | 0.27 | <0.00001 | 0.05 | 0.256442 | 0.42 | <0.00001 | -0.05 | 0.503853 | 0.37 | <0.00001 | 0.245 |
| *ATIC* | 0.35 | 0.000026 | 0.26 | 0.000013 | 0.29 | <0.00001 | 0.36 | 0.000104 | 0.34 | <0.00001 | 0.27 | 0.00013 | 0.312 |
| *PRKAG1* | 0.33 | 0.000077 | 0.27 | <0.00001 | 0.44 | <0.00001 | 0.18 | 0.058702 | 0.44 | <0.00001 | 0.29 | 0.000037 | 0.325 |
| *PAICS* | 0.55 | <0.00001 | 0.55 | <0.00001 | 0.33 | <0.00001 | 0.5 | <0.00001 | 0.41 | <0.00001 | 0.39 | <0.00001 | 0.455 |
| *MCM5* | 0.62 | <0.00001 | 0.46 | <0.00001 | 0.32 | <0.00001 | 0.58 | <0.00001 | 0.61 | <0.00001 | 0.53 | <0.00001 | 0.52 |
| #Sample | 138 | | 275 | | 517 | | 111 | | 181 | | 196 | | n.a. |

NOTE: LUAD, lung adenocarcinoma; R, Pearson correlation coefficient.

Table S5.

Whole transcriptome analysis for differential gene expression in H1975 cells treated with AICAR. N=508 genes.

| Gene name | Description | log2(fc) | P value |
| --- | --- | --- | --- |
| HAPLN2 | hyaluronan and proteoglycan link protein 2 | -9.40 | 3.41E-04 |
| SLC26A8 | solute carrier family 26 member 8 | -7.82 | 8.38E-04 |
| ATOH8 | atonal bHLH transcription factor 8 | -7.78 | 3.81E-05 |
| MAP6 | microtubule associated protein 6 | -7.34 | 3.38E-03 |
| SIM2 | SIM bHLH transcription factor 2 | -7.10 | 2.05E-03 |
| PDE11A | phosphodiesterase 11A | -6.34 | 3.69E-03 |
| AC093899 | novel protein | -4.55 | 1.17E-03 |
| ADRB1 | adrenoceptor beta 1 | -4.48 | 5.88E-10 |
| TUBA4B | tubulin alpha 4b | -3.43 | 3.62E-03 |
| RNF222 | ring finger protein 222 | -3.36 | 5.24E-04 |
| SP5 | Sp5 transcription factor | -3.22 | 5.74E-05 |
| HSPA6 | heat shock protein family A (Hsp70) member 6 | -3.16 | 2.78E-05 |
| NOG | noggin | -3.03 | 7.26E-08 |
| SPRY1 | sprouty RTK signaling antagonist 1 | -2.90 | 4.06E-19 |
| DUSP2 | dual specificity phosphatase 2 | -2.86 | 1.80E-42 |
| IL12A | interleukin 12A | -2.56 | 1.35E-03 |
| ZBTB32 | zinc finger and BTB domain containing 32 | -2.53 | 7.59E-08 |
| DUSP6 | dual specificity phosphatase 6 | -2.50 | 5.60E-07 |
| DEPP1 | DEPP1 autophagy regulator | -2.42 | 1.00E-12 |
| VANGL2 | VANGL planar cell polarity protein 2 | -2.37 | 2.31E-03 |
| KCNJ12 | potassium inwardly rectifying channel subfamily J member 12 | -2.36 | 5.52E-06 |
| KRTAP2-3 | keratin associated protein 2-3 | -2.32 | 8.41E-04 |
| TTC34 | tetratricopeptide repeat domain 34 | -2.31 | 1.27E-05 |
| CSF2 | colony stimulating factor 2 | -2.28 | 3.65E-32 |
| ACSM6 | acyl-CoA synthetase medium chain family member 6 | -2.26 | 1.51E-03 |
| DDIT3 | DNA damage inducible transcript 3 | -2.25 | 5.60E-31 |
| TRAF1 | TNF receptor associated factor 1 | -2.22 | 7.59E-61 |
| BDNF | brain derived neurotrophic factor | -2.14 | 5.22E-06 |
| PTGER4 | prostaglandin E receptor 4 | -2.11 | 4.13E-18 |
| KIAA1755 | KIAA1755 | -2.05 | 4.74E-05 |
| PLK3 | polo like kinase 3 | -2.01 | 3.85E-32 |
| PLK2 | polo like kinase 2 | -2.01 | 8.38E-57 |
| CHAC1 | ChaC glutathione specific gamma-glutamylcyclotransferase 1 | -2.01 | 5.53E-05 |
| SGK1 | serum/glucocorticoid regulated kinase 1 | -2.00 | 4.42E-29 |
| EDN1 | endothelin 1 | -2.00 | 1.80E-05 |
| CXCL8 | C-X-C motif chemokine ligand 8 | -1.98 | 3.71E-16 |
| SOCS1 | suppressor of cytokine signaling 1 | -1.98 | 1.96E-12 |
| FUT1 | fucosyltransferase 1 (H blood group) | -1.95 | 1.03E-23 |
| SKIDA1 | SKI/DACH domain containing 1 | -1.95 | 9.62E-08 |
| DKK1 | dickkopf WNT signaling pathway inhibitor 1 | -1.95 | 4.62E-99 |
| IL11 | interleukin 11 | -1.94 | 1.25E-29 |
| PPP1R3C | protein phosphatase 1 regulatory subunit 3C | -1.93 | 9.07E-14 |
| ST20-MTHFS | ST20-MTHFS readthrough | -1.92 | 2.18E-04 |
| ZNF572 | zinc finger protein 572 | -1.90 | 3.41E-13 |
| SPRY4 | sprouty RTK signaling antagonist 4 | -1.89 | 4.13E-21 |
| CD274 | CD274 molecule | -1.87 | 6.50E-33 |
| ACAP1 | ArfGAP with coiled-coil, ankyrin repeat and PH domains 1 | -1.84 | 7.08E-15 |
| PPP1R10 | protein phosphatase 1 regulatory subunit 10 | -1.84 | 4.79E-79 |
| EXTL1 | exostosin like glycosyltransferase 1 | -1.82 | 2.92E-03 |
| IER5L | immediate early response 5 like | -1.78 | 1.17E-55 |
| CLDN14 | claudin 14 | -1.78 | 1.75E-03 |
| ZIC5 | Zic family member 5 | -1.76 | 6.38E-12 |
| RGS16 | regulator of G protein signaling 16 | -1.76 | 6.23E-05 |
| EDN2 | endothelin 2 | -1.75 | 7.05E-10 |
| GJA3 | gap junction protein alpha 3 | -1.74 | 2.94E-03 |
| UFSP1 | UFM1 specific peptidase 1 (inactive) | -1.73 | 1.02E-19 |
| ANGPTL4 | angiopoietin like 4 | -1.72 | 5.41E-35 |
| MOB3C | MOB kinase activator 3C | -1.71 | 1.36E-06 |
| SOX21 | SRY-box transcription factor 21 | -1.71 | 3.94E-17 |
| HAS3 | hyaluronan synthase 3 | -1.71 | 2.23E-50 |
| ARL4D | ADP ribosylation factor like GTPase 4D | -1.70 | 2.80E-11 |
| PHETA2 | PH domain containing endocytic trafficking adaptor 2 | -1.67 | 4.61E-18 |
| LSAMP | limbic system associated membrane protein | -1.66 | 1.95E-08 |
| ADRB2 | adrenoceptor beta 2 | -1.66 | 1.86E-29 |
| PPP1R3D | protein phosphatase 1 regulatory subunit 3D | -1.65 | 4.92E-17 |
| VILL | villin like | -1.64 | 7.11E-04 |
| KCNK5 | potassium two pore domain channel subfamily K member 5 | -1.63 | 3.61E-21 |
| GATA6 | GATA binding protein 6 | -1.63 | 1.48E-14 |
| SOCS2 | suppressor of cytokine signaling 2 | -1.62 | 8.75E-04 |
| IER3 | immediate early response 3 | -1.61 | 1.83E-41 |
| ZSWIM3 | zinc finger SWIM-type containing 3 | -1.60 | 7.11E-10 |
| ZNF764 | zinc finger protein 764 | -1.60 | 1.61E-08 |
| NTN3 | netrin 3 | -1.59 | 1.09E-05 |
| KLHDC7A | kelch domain containing 7A | -1.59 | 1.14E-03 |
| NABP1 | nucleic acid binding protein 1 | -1.57 | 2.34E-44 |
| NAGS | N-acetylglutamate synthase | -1.57 | 1.94E-13 |
| CACNG8 | calcium voltage-gated channel auxiliary subunit gamma 8 | -1.57 | 1.72E-13 |
| CCL2 | C-C motif chemokine ligand 2 | -1.55 | 6.54E-04 |
| SIX1 | SIX homeobox 1 | -1.55 | 8.92E-12 |
| ZNF48 | zinc finger protein 48 | -1.54 | 5.48E-25 |
| BAMBI | BMP and activin membrane bound inhibitor | -1.54 | 1.01E-04 |
| LCMT2 | leucine carboxyl methyltransferase 2 | -1.54 | 2.55E-24 |
| DLX3 | distal-less homeobox 3 | -1.53 | 1.31E-16 |
| THY1 | Thy-1 cell surface antigen | -1.50 | 2.78E-05 |
| IQCC | IQ motif containing C | -1.50 | 6.26E-08 |
| ASB2 | ankyrin repeat and SOCS box containing 2 | -1.50 | 1.61E-04 |
| ZNF792 | zinc finger protein 792 | -1.50 | 3.14E-03 |
| DDIT4 | DNA damage inducible transcript 4 | -1.47 | 2.42E-36 |
| ZKSCAN7 | zinc finger with KRAB and SCAN domains 7 | -1.47 | 1.51E-03 |
| SPIN2A | spindlin family member 2A | -1.47 | 3.22E-04 |
| HAS2 | hyaluronan synthase 2 | -1.46 | 2.36E-07 |
| RNF227 | ring finger protein 227 | -1.46 | 3.80E-07 |
| MPP3 | membrane palmitoylated protein 3 | -1.45 | 6.19E-16 |
| ZNF319 | zinc finger protein 319 | -1.45 | 3.88E-15 |
| ZNF691 | zinc finger protein 691 | -1.45 | 3.52E-07 |
| ARL14 | ADP ribosylation factor like GTPase 14 | -1.44 | 8.28E-07 |
| PDGFB | platelet derived growth factor subunit B | -1.43 | 8.97E-28 |
| RRAD | RRAD, Ras related glycolysis inhibitor and calcium channel regulator | -1.43 | 1.60E-12 |
| USP27X | ubiquitin specific peptidase 27 X-linked | -1.43 | 1.12E-06 |
| NUTM1 | NUT midline carcinoma family member 1 | -1.43 | 3.38E-03 |
| BCAN | brevican | -1.42 | 8.58E-10 |
| NHLRC1 | NHL repeat containing E3 ubiquitin protein ligase 1 | -1.42 | 1.08E-04 |
| CCDC71L | coiled-coil domain containing 71 like | -1.42 | 2.50E-28 |
| ZNF469 | zinc finger protein 469 | -1.41 | 2.69E-34 |
| RAB7B | RAB7B, member RAS oncogene family | -1.41 | 6.74E-09 |
| IL24 | interleukin 24 | -1.39 | 9.47E-12 |
| GPR82 | G protein-coupled receptor 82 | -1.39 | 1.74E-03 |
| C6orf141 | chromosome 6 open reading frame 141 | -1.38 | 2.19E-20 |
| TENT5B | terminal nucleotidyltransferase 5B | -1.38 | 6.84E-12 |
| TRIB1 | tribbles pseudokinase 1 | -1.37 | 1.63E-33 |
| CCDC200 | coiled-coil domain containing 200 | -1.37 | 3.04E-04 |
| HEXIM2 | HEXIM P-TEFb complex subunit 2 | -1.35 | 8.10E-05 |
| GVQW3 | GVQW motif containing 3 | -1.35 | 4.21E-09 |
| RASGEF1B | RasGEF domain family member 1B | -1.35 | 3.30E-03 |
| C1orf74 | chromosome 1 open reading frame 74 | -1.35 | 1.39E-12 |
| KCNA7 | potassium voltage-gated channel subfamily A member 7 | -1.34 | 8.38E-10 |
| CD83 | CD83 molecule | -1.33 | 3.01E-05 |
| TNFSF15 | TNF superfamily member 15 | -1.33 | 2.84E-04 |
| RHOV | ras homolog family member V | -1.32 | 2.32E-10 |
| ZNF324B | zinc finger protein 324B | -1.32 | 2.34E-18 |
| BCL6B | BCL6B transcription repressor | -1.32 | 1.83E-03 |
| KCNJ18 | potassium inwardly rectifying channel subfamily J member 18 | -1.31 | 2.92E-08 |
| ZBTB3 | zinc finger and BTB domain containing 3 | -1.31 | 1.93E-06 |
| MFSD2A | major facilitator superfamily domain containing 2A | -1.30 | 7.89E-08 |
| C8orf58 | chromosome 8 open reading frame 58 | -1.29 | 1.13E-10 |
| ZBED8 | zinc finger BED-type containing 8 | -1.29 | 6.81E-08 |
| ZNF697 | zinc finger protein 697 | -1.28 | 6.85E-19 |
| GPR3 | G protein-coupled receptor 3 | -1.28 | 4.06E-04 |
| CEBPD | CCAAT enhancer binding protein delta | -1.28 | 5.10E-18 |
| SOX9 | SRY-box transcription factor 9 | -1.26 | 1.34E-34 |
| TNFAIP3 | TNF alpha induced protein 3 | -1.25 | 1.16E-30 |
| ZNF114 | zinc finger protein 114 | -1.24 | 5.02E-30 |
| DUSP4 | dual specificity phosphatase 4 | -1.24 | 1.14E-19 |
| FUT2 | fucosyltransferase 2 | -1.23 | 6.89E-14 |
| FSCN2 | fascin actin-bundling protein 2, retinal | -1.23 | 3.34E-07 |
| ADAMTS15 | ADAM metallopeptidase with thrombospondin type 1 motif 15 | -1.22 | 5.41E-19 |
| H3C13 | H3 clustered histone 13 | -1.22 | 3.99E-23 |
| FRAT1 | FRAT regulator of WNT signaling pathway 1 | -1.20 | 2.47E-03 |
| AL391987 |  | -1.20 | 2.05E-04 |
| DDX28 | DEAD-box helicase 28 | -1.20 | 2.62E-24 |
| SH3TC1 | SH3 domain and tetratricopeptide repeats 1 | -1.20 | 7.29E-07 |
| NFKBIE | NFKB inhibitor epsilon | -1.20 | 2.03E-11 |
| MAFF | MAF bZIP transcription factor F | -1.19 | 1.68E-18 |
| MCIDAS | multiciliate differentiation and DNA synthesis associated cell cycle protein | -1.19 | 1.53E-05 |
| SOWAHC | sosondowah ankyrin repeat domain family member C | -1.19 | 1.88E-46 |
| ANKRD33 | ankyrin repeat domain 33 | -1.19 | 4.47E-14 |
| HPS6 | HPS6 biogenesis of lysosomal organelles complex 2 subunit 3 | -1.18 | 1.93E-21 |
| SOCS3 | suppressor of cytokine signaling 3 | -1.18 | 1.80E-14 |
| PLEKHG4B | pleckstrin homology and RhoGEF domain containing G4B | -1.18 | 1.53E-06 |
| BCL2 | BCL2 apoptosis regulator | -1.17 | 1.65E-04 |
| LENG9 | leukocyte receptor cluster member 9 | -1.17 | 6.39E-18 |
| HS3ST1 | heparan sulfate-glucosamine 3-sulfotransferase 1 | -1.17 | 8.07E-06 |
| TMEM79 | transmembrane protein 79 | -1.17 | 2.22E-08 |
| CISH | cytokine inducible SH2 containing protein | -1.17 | 1.78E-05 |
| MOCS3 | molybdenum cofactor synthesis 3 | -1.17 | 2.56E-19 |
| ENC1 | ectodermal-neural cortex 1 | -1.16 | 8.56E-39 |
| BIRC3 | baculoviral IAP repeat containing 3 | -1.16 | 3.19E-07 |
| NKX2-8 | NK2 homeobox 8 | -1.16 | 2.01E-05 |
| FAM43A | family with sequence similarity 43 member A | -1.16 | 5.19E-05 |
| HARBI1 | harbinger transposase derived 1 | -1.16 | 3.79E-05 |
| CBARP | CACN subunit beta associated regulatory protein | -1.15 | 8.22E-06 |
| CCDC9B | coiled-coil domain containing 9B | -1.15 | 1.98E-16 |
| KLF4 | Kruppel like factor 4 | -1.14 | 9.72E-24 |
| PLK5 | polo like kinase 5 (inactive) | -1.14 | 3.26E-17 |
| FOXA2 | forkhead box A2 | -1.14 | 5.04E-07 |
| FOXL1 | forkhead box L1 | -1.13 | 3.78E-11 |
| ZNF696 | zinc finger protein 696 | -1.13 | 1.30E-09 |
| CCNE2 | cyclin E2 | -1.13 | 2.74E-11 |
| PIK3CD | phosphatidylinositol-4,5-bisphosphate 3-kinase catalytic subunit delta | -1.13 | 4.61E-12 |
| FJX1 | four-jointed box kinase 1 | -1.13 | 2.99E-16 |
| JRKL | JRK like | -1.13 | 1.73E-10 |
| OSGIN1 | oxidative stress induced growth inhibitor 1 | -1.12 | 2.76E-12 |
| ZNF70 | zinc finger protein 70 | -1.12 | 2.44E-11 |
| PHLDA1 | pleckstrin homology like domain family A member 1 | -1.11 | 7.47E-27 |
| CEBPB | CCAAT enhancer binding protein beta | -1.11 | 2.51E-19 |
| CLDN1 | claudin 1 | -1.11 | 1.15E-05 |
| F3 | coagulation factor III, tissue factor | -1.11 | 2.67E-25 |
| TNK1 | tyrosine kinase non receptor 1 | -1.11 | 1.30E-22 |
| FADD | Fas associated via death domain | -1.10 | 1.45E-29 |
| SSC5D | scavenger receptor cysteine rich family member with 5 domains | -1.10 | 1.67E-04 |
| IER2 | immediate early response 2 | -1.10 | 7.10E-30 |
| TRIM46 | tripartite motif containing 46 | -1.09 | 1.30E-03 |
| SLC9A2 | solute carrier family 9 member A2 | -1.09 | 7.31E-04 |
| PHF23 | PHD finger protein 23 | -1.09 | 7.01E-27 |
| FRAT2 | FRAT regulator of WNT signaling pathway 2 | -1.09 | 1.29E-16 |
| LHX1 | LIM homeobox 1 | -1.09 | 6.82E-05 |
| NFKBID | NFKB inhibitor delta | -1.08 | 9.74E-07 |
| G0S2 | G0/G1 switch 2 | -1.08 | 1.21E-09 |
| AMIGO2 | adhesion molecule with Ig like domain 2 | -1.08 | 8.08E-18 |
| GADD45A | growth arrest and DNA damage inducible alpha | -1.08 | 5.18E-11 |
| ZFP36L1 | ZFP36 ring finger protein like 1 | -1.08 | 2.56E-32 |
| MYO1F | myosin IF | -1.07 | 3.26E-05 |
| ZFP36L2 | ZFP36 ring finger protein like 2 | -1.07 | 3.68E-24 |
| KRBA1 | KRAB-A domain containing 1 | -1.07 | 1.25E-07 |
| BORCS6 | BLOC-1 related complex subunit 6 | -1.06 | 1.70E-08 |
| KIF25 | kinesin family member 25 | -1.06 | 2.22E-04 |
| METTL18 | methyltransferase like 18 | -1.06 | 3.92E-05 |
| CYREN | cell cycle regulator of NHEJ | -1.04 | 6.22E-17 |
| SHISAL1 | shisa like 1 | -1.04 | 1.09E-05 |
| AC092835 | novel C2H2 type zinc finger protein | -1.04 | 2.36E-05 |
| ZSWIM4 | zinc finger SWIM-type containing 4 | -1.04 | 2.87E-08 |
| DSEL | dermatan sulfate epimerase like | -1.03 | 6.10E-21 |
| TIGD5 | tigger transposable element derived 5 | -1.03 | 2.71E-10 |
| ZNF629 | zinc finger protein 629 | -1.02 | 7.53E-04 |
| HIC1 | HIC ZBTB transcriptional repressor 1 | -1.01 | 4.92E-10 |
| PTCH1 | patched 1 | -1.01 | 3.69E-12 |
| KMT5C | lysine methyltransferase 5C | -1.01 | 3.42E-11 |
| IRF1 | interferon regulatory factor 1 | -1.01 | 5.87E-24 |
| SMIM5 | small integral membrane protein 5 | -1.01 | 4.60E-04 |
| RIMBP3 | RIMS binding protein 3 | -1.00 | 1.30E-03 |
| SESN2 | sestrin 2 | -1.00 | 4.83E-11 |
| ACTL10 | actin like 10 | -1.00 | 1.21E-05 |
| ZC3H6 | zinc finger CCCH-type containing 6 | 1.00 | 3.49E-05 |
| ZNF547 | zinc finger protein 547 | 1.01 | 5.42E-07 |
| NAIP | NLR family apoptosis inhibitory protein | 1.02 | 1.12E-04 |
| NUDT17 | nudix hydrolase 17 | 1.02 | 3.49E-03 |
| GCSAM | germinal center associated signaling and motility | 1.04 | 1.14E-04 |
| AMZ1 | archaelysin family metallopeptidase 1 | 1.05 | 3.26E-05 |
| SYT15 | synaptotagmin 15 | 1.05 | 9.41E-09 |
| GNAL | G protein subunit alpha L | 1.05 | 5.27E-04 |
| ARL10 | ADP ribosylation factor like GTPase 10 | 1.05 | 2.20E-03 |
| FAM71F2 | family with sequence similarity 71 member F2 | 1.06 | 3.05E-03 |
| MARCHF1 | membrane associated ring-CH-type finger 1 | 1.06 | 4.07E-06 |
| STKLD1 | serine/threonine kinase like domain containing 1 | 1.06 | 5.41E-04 |
| CLEC18A | C-type lectin domain family 18 member A | 1.06 | 2.48E-03 |
| ATP8A1 | ATPase phospholipid transporting 8A1 | 1.07 | 6.35E-05 |
| RNASE1 | ribonuclease A family member 1, pancreatic | 1.08 | 2.21E-04 |
| NFE2L3 | nuclear factor, erythroid 2 like 3 | 1.09 | 8.13E-28 |
| ITGB1BP2 | integrin subunit beta 1 binding protein 2 | 1.10 | 2.66E-05 |
| PCF11 | PCF11 cleavage and polyadenylation factor subunit | 1.10 | 6.75E-20 |
| NECTIN4 | nectin cell adhesion molecule 4 | 1.10 | 1.38E-08 |
| KLF11 | Kruppel like factor 11 | 1.10 | 1.66E-13 |
| IL12RB2 | interleukin 12 receptor subunit beta 2 | 1.11 | 7.97E-06 |
| DIO3 | iodothyronine deiodinase 3 | 1.11 | 3.78E-03 |
| TSC22D3 | TSC22 domain family member 3 | 1.12 | 2.72E-06 |
| RHOB | ras homolog family member B | 1.12 | 1.83E-26 |
| ARRDC4 | arrestin domain containing 4 | 1.12 | 7.28E-13 |
| ZBTB43 | zinc finger and BTB domain containing 43 | 1.13 | 1.16E-20 |
| SLC25A18 | solute carrier family 25 member 18 | 1.13 | 1.82E-05 |
| FSD2 | fibronectin type III and SPRY domain containing 2 | 1.15 | 4.12E-04 |
| C19orf18 | chromosome 19 open reading frame 18 | 1.15 | 4.24E-05 |
| IGIP | IgA inducing protein | 1.16 | 3.26E-05 |
| SLC16A4 | solute carrier family 16 member 4 | 1.17 | 9.02E-06 |
| VTN;SEBOX | vitronectin;SEBOX homeobox | 1.20 | 3.91E-06 |
| CFAP70 | cilia and flagella associated protein 70 | 1.22 | 1.59E-03 |
| CCDC39 | coiled-coil domain containing 39 | 1.23 | 1.26E-04 |
| ZBTB34 | zinc finger and BTB domain containing 34 | 1.23 | 1.71E-15 |
| KIF26B | kinesin family member 26B | 1.27 | 9.49E-04 |
| STIMATE-MUSTN1 | STIMATE-MUSTN1 readthrough | 1.28 | 3.32E-05 |
| ADCY5 | adenylate cyclase 5 | 1.29 | 1.02E-03 |
| TRIM74 | tripartite motif containing 74 | 1.29 | 3.31E-03 |
| MYRF | myelin regulatory factor | 1.30 | 3.64E-07 |
| IFI27 | interferon alpha inducible protein 27 | 1.31 | 8.32E-08 |
| GPX2 | glutathione peroxidase 2 | 1.31 | 1.81E-05 |
| SPON1 | spondin 1 | 1.31 | 8.09E-08 |
| GPLD1 | glycosylphosphatidylinositol specific phospholipase D1 | 1.32 | 4.28E-04 |
| NOSTRIN | nitric oxide synthase trafficking | 1.33 | 3.57E-04 |
| THSD1 | thrombospondin type 1 domain containing 1 | 1.34 | 3.17E-05 |
| CTSK | cathepsin K | 1.34 | 7.29E-07 |
| PSPN | persephin | 1.34 | 2.82E-10 |
| SMPD3 | sphingomyelin phosphodiesterase 3 | 1.34 | 3.05E-07 |
| NDUFA4L2 | NDUFA4 mitochondrial complex associated like 2 | 1.34 | 5.78E-04 |
| IL20RB | interleukin 20 receptor subunit beta | 1.34 | 2.68E-05 |
| TAS2R5 | taste 2 receptor member 5 | 1.35 | 9.21E-04 |
| LSM8 | LSM8 homolog, U6 small nuclear RNA associated | 1.36 | 7.12E-25 |
| PHEX | phosphate regulating endopeptidase homolog X-linked | 1.36 | 1.28E-04 |
| KCNK12 | potassium two pore domain channel subfamily K member 12 | 1.37 | 2.01E-10 |
| RGMA | repulsive guidance molecule BMP co-receptor a | 1.38 | 5.43E-04 |
| SPOCK2 | SPARC (osteonectin), cwcv and kazal like domains proteoglycan 2 | 1.38 | 2.37E-07 |
| CD160 | CD160 molecule | 1.38 | 9.39E-04 |
| PLPPR3 | phospholipid phosphatase related 3 | 1.39 | 3.17E-06 |
| TAS2R4 | taste 2 receptor member 4 | 1.40 | 1.55E-07 |
| SIGLEC15 | sialic acid binding Ig like lectin 15 | 1.43 | 5.42E-06 |
| CSRP2 | cysteine and glycine rich protein 2 | 1.43 | 4.07E-04 |
| GRTP1 | growth hormone regulated TBC protein 1 | 1.44 | 1.73E-06 |
| CLDN5 | claudin 5 | 1.44 | 2.01E-06 |
| PDZK1 | PDZ domain containing 1 | 1.45 | 1.33E-03 |
| MCMDC2 | minichromosome maintenance domain containing 2 | 1.45 | 1.25E-13 |
| KCNB1 | potassium voltage-gated channel subfamily B member 1 | 1.47 | 4.04E-04 |
| ADAM20 | ADAM metallopeptidase domain 20 | 1.49 | 7.91E-04 |
| CEL | carboxyl ester lipase | 1.49 | 9.01E-06 |
| NLRC3 | NLR family CARD domain containing 3 | 1.51 | 3.52E-05 |
| YPEL1 | yippee like 1 | 1.51 | 3.26E-03 |
| KLHL3 | kelch like family member 3 | 1.52 | 3.69E-06 |
| MSANTD1 | Myb/SANT DNA binding domain containing 1 | 1.54 | 5.04E-06 |
| MEIOC | meiosis specific with coiled-coil domain | 1.55 | 5.10E-08 |
| MAPK10 | mitogen-activated protein kinase 10 | 1.56 | 1.67E-03 |
| CA14 | carbonic anhydrase 14 | 1.58 | 3.36E-05 |
| MMRN2 | multimerin 2 | 1.59 | 9.58E-04 |
| RHBDL1 | rhomboid like 1 | 1.60 | 4.84E-04 |
| CLEC18C | C-type lectin domain family 18 member C | 1.61 | 1.31E-05 |
| TMEM269 | transmembrane protein 269 | 1.61 | 2.68E-03 |
| PIK3IP1 | phosphoinositide-3-kinase interacting protein 1 | 1.63 | 2.02E-04 |
| GRIN3A | glutamate ionotropic receptor NMDA type subunit 3A | 1.63 | 7.55E-08 |
| DYRK4 | dual specificity tyrosine phosphorylation regulated kinase 4 | 1.63 | 1.54E-07 |
| ANKDD1B | ankyrin repeat and death domain containing 1B | 1.66 | 1.99E-04 |
| RRH | retinal pigment epithelium-derived rhodopsin homolog | 1.66 | 4.81E-05 |
| GCNA | germ cell nuclear acidic peptidase | 1.68 | 2.41E-12 |
| CDKL4 | cyclin dependent kinase like 4 | 1.69 | 2.55E-03 |
| UCN | urocortin | 1.70 | 5.02E-04 |
| CD302;LY75 | CD302 molecule;lymphocyte antigen 75 | 1.71 | 1.99E-05 |
| RUNDC3A | RUN domain containing 3A | 1.71 | 7.20E-07 |
| TSSK4 | testis specific serine kinase 4 | 1.72 | 1.35E-04 |
| ADAMTSL4 | ADAMTS like 4 | 1.75 | 2.55E-16 |
| BNIPL | BCL2 interacting protein like | 1.77 | 2.00E-05 |
| KCNS1 | potassium voltage-gated channel modifier subfamily S member 1 | 1.77 | 3.76E-06 |
| FBN1 | fibrillin 1 | 1.77 | 1.42E-04 |
| WDR86 | WD repeat domain 86 | 1.77 | 1.24E-03 |
| C1QTNF9B | C1q and TNF related 9B | 1.78 | 6.54E-06 |
| ZFHX2 | zinc finger homeobox 2 | 1.79 | 1.38E-10 |
| ADGRA2 | adhesion G protein-coupled receptor A2 | 1.80 | 3.00E-06 |
| TMEM81 | transmembrane protein 81 | 1.82 | 7.43E-09 |
| LCT | lactase | 1.82 | 3.44E-11 |
| DCAF4L1 | DDB1 and CUL4 associated factor 4 like 1 | 1.84 | 2.56E-08 |
| RAB40AL | RAB40A like | 1.87 | 1.34E-03 |
| NAALAD2 | N-acetylated alpha-linked acidic dipeptidase 2 | 1.87 | 9.65E-08 |
| KCNH3 | potassium voltage-gated channel subfamily H member 3 | 1.88 | 2.92E-04 |
| GCNT3 | glucosaminyl (N-acetyl) transferase 3, mucin type | 1.91 | 9.97E-05 |
| MCC | MCC regulator of WNT signaling pathway | 1.93 | 5.11E-10 |
| LDLRAD4 | low density lipoprotein receptor class A domain containing 4 | 1.94 | 1.37E-06 |
| CLEC18B | C-type lectin domain family 18 member B | 1.95 | 4.97E-08 |
| P2RX2 | purinergic receptor P2X 2 | 1.97 | 1.10E-04 |
| SLC10A5 | solute carrier family 10 member 5 | 1.98 | 1.80E-12 |
| C11orf42 | chromosome 11 open reading frame 42 | 2.03 | 5.24E-04 |
| DNAAF1 | dynein axonemal assembly factor 1 | 2.04 | 6.02E-12 |
| LRRN4 | leucine rich repeat neuronal 4 | 2.06 | 1.87E-07 |
| VWF | von Willebrand factor | 2.09 | 1.51E-03 |
| GNRH1 | gonadotropin releasing hormone 1 | 2.09 | 2.52E-05 |
| CNTN2 | contactin 2 | 2.10 | 2.57E-08 |
| TMEM236 | transmembrane protein 236 | 2.13 | 6.45E-19 |
| SLC22A1 | solute carrier family 22 member 1 | 2.13 | 5.07E-11 |
| PDZD2 | PDZ domain containing 2 | 2.14 | 1.04E-14 |
| CAMK4 | calcium/calmodulin dependent protein kinase IV | 2.15 | 1.83E-04 |
| IMPG2 | interphotoreceptor matrix proteoglycan 2 | 2.16 | 5.55E-08 |
| CMYA5 | cardiomyopathy associated 5 | 2.19 | 5.49E-04 |
| FAM229A | family with sequence similarity 229 member A | 2.20 | 6.98E-16 |
| TP53AIP1 | tumor protein p53 regulated apoptosis inducing protein 1 | 2.21 | 3.45E-06 |
| RFTN2 | raftlin family member 2 | 2.22 | 6.14E-20 |
| RUNDC3B | RUN domain containing 3B | 2.23 | 1.06E-03 |
| PNLDC1 | PARN like, ribonuclease domain containing 1 | 2.25 | 9.90E-07 |
| PCDH12 | protocadherin 12 | 2.25 | 2.44E-13 |
| DRP2 | dystrophin related protein 2 | 2.29 | 1.82E-08 |
| MUC20 | mucin 20, cell surface associated | 2.32 | 7.35E-08 |
| PKD1L1 | polycystin 1 like 1, transient receptor potential channel interacting | 2.32 | 1.44E-10 |
| ODF3 | outer dense fiber of sperm tails 3 | 2.33 | 2.68E-06 |
| ARR3 | arrestin 3 | 2.36 | 2.29E-04 |
| MASP2 | mannan binding lectin serine peptidase 2 | 2.36 | 2.76E-03 |
| RNF225 | ring finger protein 225 | 2.36 | 4.65E-11 |
| GZMM | granzyme M | 2.37 | 8.38E-05 |
| KCNAB1 | potassium voltage-gated channel subfamily A member regulatory beta subunit 1 | 2.38 | 1.27E-17 |
| PROX2 | prospero homeobox 2 | 2.39 | 2.41E-13 |
| MYO1A | myosin IA | 2.39 | 1.20E-06 |
| SCG5 | secretogranin V | 2.44 | 1.14E-09 |
| ABCG8 | ATP binding cassette subfamily G member 8 | 2.46 | 4.45E-05 |
| SFTPB | surfactant protein B | 2.46 | 4.04E-11 |
| OVGP1 | oviductal glycoprotein 1 | 2.47 | 1.53E-11 |
| MROH2A | maestro heat like repeat family member 2A | 2.47 | 1.64E-05 |
| CCDC17 | coiled-coil domain containing 17 | 2.49 | 5.89E-28 |
| POPDC2 | popeye domain containing 2 | 2.51 | 3.49E-10 |
| GUCA1B | guanylate cyclase activator 1B | 2.53 | 1.07E-13 |
| SLIT1 | slit guidance ligand 1 | 2.53 | 2.08E-05 |
| ADAM22 | ADAM metallopeptidase domain 22 | 2.55 | 3.54E-06 |
| IL3RA | interleukin 3 receptor subunit alpha | 2.56 | 4.49E-07 |
| TEX35 | testis expressed 35 | 2.57 | 2.25E-03 |
| C9orf131 | chromosome 9 open reading frame 131 | 2.58 | 8.07E-12 |
| ZC3H12D | zinc finger CCCH-type containing 12D | 2.58 | 1.26E-06 |
| SYN3 | synapsin III | 2.60 | 3.62E-05 |
| VIL1 | villin 1 | 2.63 | 7.60E-04 |
| CRHR2 | corticotropin releasing hormone receptor 2 | 2.63 | 6.21E-08 |
| CYP26C1 | cytochrome P450 family 26 subfamily C member 1 | 2.63 | 1.99E-06 |
| RASGRP3 | RAS guanyl releasing protein 3 | 2.64 | 1.38E-07 |
| HCLS1 | hematopoietic cell-specific Lyn substrate 1 | 2.65 | 1.27E-03 |
| CCDC38 | coiled-coil domain containing 38 | 2.66 | 2.12E-04 |
| IDI2 | isopentenyl-diphosphate delta isomerase 2 | 2.69 | 2.18E-05 |
| NR4A3 | nuclear receptor subfamily 4 group A member 3 | 2.69 | 2.21E-05 |
| NECAB1 | N-terminal EF-hand calcium binding protein 1 | 2.69 | 6.02E-05 |
| APOE | apolipoprotein E | 2.70 | 7.04E-07 |
| PECAM1 | platelet and endothelial cell adhesion molecule 1 | 2.72 | 3.05E-03 |
| KLRG2 | killer cell lectin like receptor G2 | 2.73 | 4.06E-06 |
| SPARCL1 | SPARC like 1 | 2.75 | 4.74E-04 |
| RAPSN | receptor associated protein of the synapse | 2.78 | 7.74E-20 |
| MSH4 | mutS homolog 4 | 2.78 | 1.44E-07 |
| LRIT3 | leucine rich repeat, Ig-like and transmembrane domains 3 | 2.78 | 4.50E-06 |
| JAM2 | junctional adhesion molecule 2 | 2.84 | 1.43E-13 |
| SUSD5 | sushi domain containing 5 | 2.85 | 1.70E-05 |
| CHRD | chordin | 2.86 | 3.48E-03 |
| C1orf162 | chromosome 1 open reading frame 162 | 2.91 | 5.00E-10 |
| PLEKHS1 | pleckstrin homology domain containing S1 | 2.95 | 2.26E-04 |
| COL6A3 | collagen type VI alpha 3 chain | 2.95 | 3.21E-06 |
| COL20A1 | collagen type XX alpha 1 chain | 2.96 | 3.34E-08 |
| TCF7L1 | transcription factor 7 like 1 | 2.97 | 2.12E-06 |
| MYOZ3 | myozenin 3 | 2.99 | 9.82E-05 |
| TCAP | titin-cap | 3.00 | 1.43E-03 |
| CCDC62 | coiled-coil domain containing 62 | 3.02 | 1.79E-17 |
| PYCARD | PYD and CARD domain containing | 3.03 | 3.53E-05 |
| THPO | thrombopoietin | 3.07 | 1.22E-03 |
| CASS4 | Cas scaffold protein family member 4 | 3.12 | 3.74E-24 |
| KCND3 | potassium voltage-gated channel subfamily D member 3 | 3.21 | 8.25E-04 |
| C2orf66 | chromosome 2 open reading frame 66 | 3.22 | 1.14E-04 |
| PKD1L3 | polycystin 1 like 3, transient receptor potential channel interacting | 3.22 | 6.34E-10 |
| SPX | spexin hormone | 3.24 | 1.68E-11 |
| CD8A | CD8a molecule | 3.24 | 2.60E-04 |
| GJA4 | gap junction protein alpha 4 | 3.27 | 1.75E-08 |
| PEX5L | peroxisomal biogenesis factor 5 like | 3.31 | 2.47E-12 |
| SNTB1 | syntrophin beta 1 | 3.31 | 2.57E-07 |
| LHFPL4 | LHFPL tetraspan subfamily member 4 | 3.35 | 3.97E-12 |
| ADAM21 | ADAM metallopeptidase domain 21 | 3.43 | 4.32E-13 |
| TTC23L | tetratricopeptide repeat domain 23 like | 3.43 | 5.00E-04 |
| C1orf100 | chromosome 1 open reading frame 100 | 3.58 | 3.64E-06 |
| PYGM | glycogen phosphorylase, muscle associated | 3.66 | 1.24E-34 |
| TEX29 | testis expressed 29 | 3.67 | 1.67E-06 |
| FBLL1 | fibrillarin like 1 | 3.77 | 2.49E-03 |
| FAM81B | family with sequence similarity 81 member B | 3.77 | 2.60E-03 |
| FAP | fibroblast activation protein alpha | 3.80 | 7.86E-05 |
| COLEC11 | collectin subfamily member 11 | 3.80 | 7.70E-20 |
| CALB1 | calbindin 1 | 3.86 | 9.75E-04 |
| CD226 | CD226 molecule | 3.87 | 2.55E-03 |
| EFCAB12 | EF-hand calcium binding domain 12 | 3.94 | 2.08E-32 |
| DRC1 | dynein regulatory complex subunit 1 | 3.98 | 1.69E-03 |
| GPR1 | G protein-coupled receptor 1 | 4.01 | 3.76E-03 |
| P2RY4 | pyrimidinergic receptor P2Y4 | 4.10 | 1.04E-03 |
| PLET1 | placenta expressed transcript 1 | 4.10 | 3.70E-05 |
| FCER1G | Fc fragment of IgE receptor Ig | 4.14 | 1.34E-04 |
| MRC1 | mannose receptor C-type 1 | 4.16 | 5.35E-05 |
| MAP3K19 | mitogen-activated protein kinase kinase kinase 19 | 4.27 | 2.49E-03 |
| UPK1A | uroplakin 1A | 4.27 | 1.92E-03 |
| CD200R1 | CD200 receptor 1 | 4.31 | 7.03E-04 |
| FPR3 | formyl peptide receptor 3 | 4.31 | 2.02E-03 |
| FNDC7 | fibronectin type III domain containing 7 | 4.37 | 2.77E-06 |
| BTLA | B and T lymphocyte associated | 4.43 | 3.33E-04 |
| BIN2 | bridging integrator 2 | 4.48 | 3.91E-06 |
| THRSP | thyroid hormone responsive | 4.51 | 8.69E-04 |
| GABRR2 | gamma-aminobutyric acid type A receptor subunit rho2 | 4.62 | 4.86E-04 |
| RNF183 | ring finger protein 183 | 4.62 | 1.94E-06 |
| MSLNL | mesothelin like | 4.64 | 3.79E-05 |
| CD163 | CD163 molecule | 4.65 | 1.41E-04 |
| ALDOB | aldolase, fructose-bisphosphate B | 4.67 | 4.10E-04 |
| AMY2B | amylase alpha 2B | 4.76 | 1.17E-05 |
| ZFP92 | ZFP92 zinc finger protein | 4.82 | 3.59E-05 |
| EXOC3L2 | exocyst complex component 3 like 2 | 4.83 | 4.77E-04 |
| SFTPA2 | surfactant protein A2 | 4.91 | 5.35E-05 |
| ITIH2 | inter-alpha-trypsin inhibitor heavy chain 2 | 4.99 | 1.97E-06 |
| TYRP1 | tyrosinase related protein 1 | 5.80 | 1.62E-05 |
| PRSS37 | serine protease 37 | 5.81 | 4.27E-08 |
| ST8SIA5 | ST8 alpha-N-acetyl-neuraminide alpha-2,8-sialyltransferase 5 | 6.10 | 3.14E-03 |
| COL1A2 | collagen type I alpha 2 chain | 6.20 | 2.95E-09 |
| PTPRO | protein tyrosine phosphatase receptor type O | 6.85 | 1.92E-05 |
| LRRK2 | leucine rich repeat kinase 2 | 6.87 | 5.91E-04 |
| TTLL7 | tubulin tyrosine ligase like 7 | 6.87 | 2.82E-03 |
| CSRNP3 | cysteine and serine rich nuclear protein 3 | 6.91 | 5.69E-04 |
| GALNTL6 | polypeptide N-acetylgalactosaminyltransferase like 6 | 7.04 | 2.42E-03 |
| GRIA4 | glutamate ionotropic receptor AMPA type subunit 4 | 7.12 | 1.58E-03 |
| PDGFRA | platelet derived growth factor receptor alpha | 7.15 | 1.37E-03 |
| DOK6 | docking protein 6 | 7.26 | 8.07E-04 |
| PCDH19 | protocadherin 19 | 7.31 | 6.81E-04 |
| PGR | progesterone receptor | 7.33 | 5.31E-05 |
| SLCO2B1 | solute carrier organic anion transporter family member 2B1 | 7.35 | 4.31E-04 |
| THBS2 | thrombospondin 2 | 7.60 | 2.19E-03 |
| TBR1 | T-box brain transcription factor 1 | 7.63 | 1.03E-04 |
| PTGER3 | prostaglandin E receptor 3 | 7.75 | 6.28E-05 |
| C7 | complement C7 | 7.75 | 3.40E-03 |
| MAP2 | microtubule associated protein 2 | 7.89 | 7.99E-06 |
| MGAM2 | maltase-glucoamylase 2 (putative) | 7.94 | 9.18E-05 |
| DMBT1 | deleted in malignant brain tumors 1 | 7.97 | 3.49E-05 |
| PTPN22 | protein tyrosine phosphatase non-receptor type 22 | 8.24 | 2.81E-03 |
| TRIM31 | tripartite motif containing 31 | 8.29 | 3.24E-03 |
| ASB4 | ankyrin repeat and SOCS box containing 4 | 8.35 | 6.82E-04 |
| TLR3 | toll like receptor 3 | 8.39 | 7.16E-05 |
| GLIS1 | GLIS family zinc finger 1 | 8.49 | 2.78E-03 |
| TG | thyroglobulin | 8.51 | 2.90E-07 |
| KCNH7 | potassium voltage-gated channel subfamily H member 7 | 8.59 | 1.23E-04 |
| NXPE1 | neurexophilin and PC-esterase domain family member 1 | 8.60 | 2.48E-04 |
| PALMD | palmdelphin | 8.68 | 1.24E-06 |
| LGR5 | leucine rich repeat containing G protein-coupled receptor 5 | 8.69 | 5.76E-05 |
| NLRP9 | NLR family pyrin domain containing 9 | 8.70 | 5.70E-04 |
| LGR6 | leucine rich repeat containing G protein-coupled receptor 6 | 8.81 | 1.00E-04 |
| TSPYL6 | TSPY like 6 | 8.89 | 1.12E-03 |
| CLGN | calmegin | 8.96 | 9.80E-04 |
| FCN3 | ficolin 3 | 8.97 | 2.97E-03 |
| SLC6A13 | solute carrier family 6 member 13 | 8.99 | 5.09E-05 |
| SLC6A4 | solute carrier family 6 member 4 | 9.05 | 9.95E-07 |
| LUM | lumican | 9.18 | 6.32E-04 |
| THEMIS | thymocyte selection associated | 9.21 | 1.56E-05 |
| CLCN1 | chloride voltage-gated channel 1 | 9.22 | 9.52E-05 |
| HGFAC | HGF activator | 9.26 | 2.83E-04 |
| HTRA4 | HtrA serine peptidase 4 | 9.26 | 1.85E-03 |
| CLEC5A | C-type lectin domain containing 5A | 9.42 | 7.09E-06 |
| SPATC1 | spermatogenesis and centriole associated 1 | 9.47 | 1.38E-03 |
| ANKRD40CL | ANKRD40 C-terminal like | 9.49 | 1.02E-05 |
| MAGEA8 | MAGE family member A8 | 9.54 | 2.53E-04 |
| TNMD | tenomodulin | 9.59 | 2.63E-03 |
| ACR | acrosin | 9.68 | 3.44E-04 |
| NKAPL | NFKB activating protein like | 9.80 | 7.44E-04 |
| OR9A4 | olfactory receptor family 9 subfamily A member 4 | 9.81 | 1.93E-04 |
| TIMP3 | TIMP metallopeptidase inhibitor 3 | 9.82 | 2.33E-07 |
| CYP26A1 | cytochrome P450 family 26 subfamily A member 1 | 10.11 | 3.81E-06 |
| CNMD | chondromodulin | 10.18 | 2.42E-04 |
| PLSCR2 | phospholipid scramblase 2 | 10.18 | 3.01E-06 |
| MIA | MIA SH3 domain containing | 10.21 | 1.17E-03 |
| CLEC20A | C-type lectin domain containing 20A | 10.28 | 1.48E-07 |
| MGAM | maltase-glucoamylase | 10.43 | 1.85E-13 |
| OR5K2 | olfactory receptor family 5 subfamily K member 2 | 10.45 | 8.92E-04 |
| A2M | alpha-2-macroglobulin | 10.48 | 5.01E-11 |
| PLA2G10 | phospholipase A2 group X | 10.59 | 2.71E-03 |
| VEGFD | vascular endothelial growth factor D | 10.76 | 5.77E-07 |
| SFTPA1 | surfactant protein A1 | 10.80 | 1.16E-07 |
| GPR25 | G protein-coupled receptor 25 | 10.88 | 3.71E-05 |
| COL3A1 | collagen type III alpha 1 chain | 11.02 | 3.89E-13 |

**Table S6.**

KEGG signaling pathway enrichment analysis in H1975 cells treated with AICAR compared with a vehicle. N =3 replicates.

| Pathway ID | Pathway name | S gene number | TS gene number | B gene number | TB gene number | P value |
| --- | --- | --- | --- | --- | --- | --- |
| ko04630 | Jak-STAT signaling pathway | 16 | 184 | 138 | 6157 | 2.90E-06 |
| ko04668 | TNF signaling pathway | 12 | 184 | 103 | 6157 | 5.04E-05 |
| ko04024 | cAMP signaling pathway | 14 | 184 | 163 | 6157 | 3.38E-04 |
| ko04933 | AGE-RAGE signaling pathway in diabetic complications | 10 | 184 | 92 | 6157 | 3.83E-04 |
| ko04080 | Neuroactive ligand-receptor interaction | 15 | 184 | 194 | 6157 | 6.42E-04 |
| ko04210 | Apoptosis | 11 | 184 | 128 | 6157 | 1.46E-03 |
| ko05146 | Amoebiasis | 8 | 184 | 79 | 6157 | 2.33E-03 |
| ko04917 | Prolactin signaling pathway | 7 | 184 | 62 | 6157 | 2.33E-03 |
| ko04973 | Carbohydrate digestion and absorption | 5 | 184 | 34 | 6157 | 3.11E-03 |
| ko05144 | Malaria | 5 | 184 | 35 | 6157 | 3.54E-03 |
| ko04742 | Taste transduction | 6 | 184 | 52 | 6157 | 4.26E-03 |
| ko04514 | Cell adhesion molecules (CAMs) | 9 | 184 | 107 | 6157 | 4.51E-03 |
| ko05133 | Pertussis | 7 | 184 | 71 | 6157 | 5.03E-03 |
| ko04924 | Renin secretion | 6 | 184 | 54 | 6157 | 5.15E-03 |
| ko05200 | Pathways in cancer | 24 | 184 | 463 | 6157 | 5.32E-03 |
| ko04930 | Type II diabetes mellitus | 5 | 184 | 41 | 6157 | 7.07E-03 |
| ko04510 | Focal adhesion | 12 | 184 | 184 | 6157 | 8.76E-03 |
| ko00500 | Starch and sucrose metabolism | 4 | 184 | 28 | 6157 | 9.01E-03 |
| ko05202 | Transcriptional misregulation in cancer | 11 | 184 | 163 | 6157 | 9.32E-03 |
| ko04610 | Complement and coagulation cascades | 6 | 184 | 61 | 6157 | 9.33E-03 |
| ko04923 | Regulation of lipolysis in adipocytes | 5 | 184 | 45 | 6157 | 1.05E-02 |
| ko04657 | IL-17 signaling pathway | 7 | 184 | 83 | 6157 | 1.16E-02 |
| ko04620 | Toll-like receptor signaling pathway | 7 | 184 | 85 | 6157 | 1.32E-02 |
| ko04215 | Apoptosis - multiple species | 4 | 184 | 32 | 6157 | 1.44E-02 |
| ko05142 | Chagas disease (American trypanosomiasis) | 7 | 184 | 90 | 6157 | 1.76E-02 |
| ko04310 | Wnt signaling pathway | 9 | 184 | 133 | 6157 | 1.77E-02 |
| ko05134 | Legionellosis | 5 | 184 | 53 | 6157 | 2.03E-02 |
| ko05033 | Nicotine addiction | 3 | 184 | 20 | 6157 | 2.06E-02 |
| ko04625 | C-type lectin receptor signaling pathway | 7 | 184 | 96 | 6157 | 2.42E-02 |
| ko05162 | Measles | 8 | 184 | 118 | 6157 | 2.45E-02 |
| ko04670 | Leukocyte transendothelial migration | 7 | 184 | 100 | 6157 | 2.95E-02 |
| ko05164 | Influenza A | 9 | 184 | 146 | 6157 | 3.03E-02 |
| ko04621 | NOD-like receptor signaling pathway | 9 | 184 | 148 | 6157 | 3.26E-02 |
| ko04151 | PI3K-Akt signaling pathway | 15 | 184 | 301 | 6157 | 3.50E-02 |
| ko04910 | Insulin signaling pathway | 8 | 184 | 128 | 6157 | 3.73E-02 |
| ko05222 | Small cell lung cancer | 6 | 184 | 88 | 6157 | 4.73E-02 |
| ko04640 | Hematopoietic cell lineage | 5 | 184 | 67 | 6157 | 4.91E-02 |
| ko00052 | Galactose metabolism | 3 | 184 | 28 | 6157 | 4.98E-02 |
| ko04975 | Fat digestion and absorption | 3 | 184 | 28 | 6157 | 4.98E-02 |

Note: Significant (p value<0.05) KEGG pathways were calculated by Hypergeometric equation. TB gene number=number of total genes; TS gene number=number of differentially expressed genes in total genes; B gene number=total number of genes in KEGG pathways; S gene number=number of differentially expressed genes in this KEGG pathway.

Table S7.

Top KEGG signaling pathways by gene set enrichment analysis in H1975 cells treated with AICAR. N =3 replicates.

| KEGG NAME | Gene number | Enrichment score | Normalized enrichment score | Normalized p value | FDR q-val | Correlation to AICAR treatment |
| --- | --- | --- | --- | --- | --- | --- |
| TNF signaling pathway | 103 | -0.5754685 | -2.3172070 | <1.0E-09 | <1.0E-09 | negative |
| Taste transduction | 52 | 0.5993102 | 1.9769673 | <1.0E-09 | 0.01168041 | positive |
| ECM receptor interaction | 71 | 0.5326455 | 1.8720856 | <1.0E-09 | 0.01233976 | positive |
| Oxidative phosphorylation | 116 | 0.4932351 | 1.8533258 | <1.0E-09 | 0.01277248 | positive |
| Transcriptional misregulation in cancer | 163 | -0.4352946 | -1.8980387 | <1.0E-09 | 0.01358726 | negative |
| Malaria | 35 | -0.5767485 | -1.9356099 | <1.0E-09 | 0.01416139 | negative |
| IL-17 signaling pathway | 83 | -0.4765815 | -1.9210117 | <1.0E-09 | 0.01482697 | negative |
| Staphylococcus aureus infection | 49 | 0.5698539 | 1.8760679 | <1.0E-09 | 0.01524310 | positive |
| Glycosphingolipid biosynthesis - lacto and neolacto series | 25 | -0.6009800 | -1.8516719 | <1.0E-09 | 0.01917320 | negative |
| Ribosome | 132 | 0.4776162 | 1.8101478 | <1.0E-09 | 0.01986750 | positive |
| Parkinson disease | 117 | 0.4674014 | 1.7671150 | <1.0E-09 | 0.02546817 | positive |
| Renin secretion | 54 | -0.4898177 | -1.7587239 | <1.0E-09 | 0.03949565 | negative |
| JAK-STAT signaling pathway | 138 | -0.3923307 | -1.6699370 | <1.0E-09 | 0.06047357 | negative |
| HERPES simplex virus 1 infection | 461 | -0.3326891 | -1.6403223 | <1.0E-09 | 0.07247353 | negative |
| MAPK signaling pathway | 264 | -0.3449815 | -1.5805273 | <1.0E-09 | 0.08211511 | negative |
| Cytokine-cytokine receptor interaction | 213 | -0.3512561 | -1.5902758 | <1.0E-09 | 0.09883131 | negative |
| Protein digestion and absorption | 65 | 0.5152535 | 1.7809000 | 0.001526718 | 0.02547104 | positive |
| NF-KAPPA B signaling pathway | 82 | -0.5125290 | -2.0019612 | 0.002932551 | 0.01140765 | negative |
| Bile secretion | 50 | 0.4957955 | 1.6532980 | 0.003149606 | 0.08878010 | positive |
| Systemic lupus erythematosus | 113 | -0.3992878 | -1.6839725 | 0.003184713 | 0.05748747 | negative |
| Apoptosis | 129 | -0.3723393 | -1.5760899 | 0.003333333 | 0.08042013 | negative |
| Asthma | 18 | 0.7203819 | 1.8816165 | 0.003424657 | 0.02016790 | positive |
| MicroRNAs in cancer | 143 | -0.3676127 | -1.5841624 | 0.003448276 | 0.08446392 | negative |
| Regulation of lipolysis in adipocytes | 45 | -0.4876932 | -1.7068236 | 0.005665722 | 0.05759150 | negative |
| Glycosaminoglycan biosynthesis heparan sulfate/heparin | 20 | -0.6055340 | -1.7045472 | 0.007751938 | 0.05240653 | negative |
| Legionellosis | 53 | -0.4327611 | -1.5687978 | 0.011235955 | 0.08092923 | negative |
| Longevity regulating pathway multiple species | 56 | -0.4299144 | -1.5901483 | 0.012048192 | 0.09185883 | negative |
| Vibrio cholerae infection | 47 | 0.4878011 | 1.5867414 | 0.012288786 | 0.09903219 | positive |
| Proteasome | 42 | 0.5111846 | 1.5934843 | 0.017432647 | 0.09916976 | positive |
| Citrate cycle (TCA cycle) | 30 | 0.5464937 | 1.6023548 | 0.017488075 | 0.09640672 | positive |
| African trypanosomiasis | 26 | -0.5166336 | -1.5885930 | 0.018518519 | 0.08761974 | negative |

Note: FDR, false discovery rate.

Table S8.

The core enrichment genes in the JAK-STAT signaling pathway by gene set enrichment analysis in H1975 cells treated with AICAR compared with vehicle treatment. N=3 replicates.

| Symbol | Rank in gene list | Rank metric score | Running enrichment score |
| --- | --- | --- | --- |
| JAK2 | 14056 | -0.427898079 | -0.38700855 |
| CSH1 | 14058 | -0.428845018 | -0.38173747 |
| MYC | 14214 | -0.469185174 | -0.38564408 |
| BCL2L1 | 14288 | -0.489939094 | -0.38413858 |
| IL22RA1 | 14292 | -0.490910858 | -0.37822124 |
| IL5 | 14321 | -0.500003457 | -0.37376216 |
| IL17D | 14418 | -0.525848985 | -0.37325564 |
| IFNAR2 | 14448 | -0.534944057 | -0.36842483 |
| PIK3R1 | 14527 | -0.560449183 | -0.3663566 |
| JAK3 | 14748 | -0.633305073 | -0.3723074 |
| EGFR | 14794 | -0.647791505 | -0.36707866 |
| IL6 | 14822 | -0.658973813 | -0.36057946 |
| CSH2 | 14884 | -0.682669461 | -0.35592255 |
| IL27RA | 14911 | -0.691033423 | -0.34896174 |
| IL4R | 14991 | -0.723320544 | -0.3449306 |
| IL7R | 15027 | -0.741480172 | -0.337908 |
| EPOR | 15067 | -0.763069272 | -0.33086833 |
| IFNLR1 | 15072 | -0.765568674 | -0.32159767 |
| CCND1 | 15217 | -0.842404246 | -0.32017085 |
| MCL1 | 15282 | -0.883360982 | -0.31320634 |
| LIF | 15499 | -1.044685483 | -0.313789 |
| PDGFA | 15765 | -1.434943795 | -0.31259754 |
| PIK3CD | 15884 | -1.745522976 | -0.29830363 |
| SOCS2 | 15891 | -1.785329103 | -0.276475 |
| CISH | 15920 | -1.87768352 | -0.2548805 |
| BCL2 | 15922 | -1.884200096 | -0.23150787 |
| IL24 | 15924 | -1.891988039 | -0.20803837 |
| IL12A | 15937 | -1.928874016 | -0.18480149 |
| SOCS3 | 15938 | -1.935699582 | -0.16072547 |
| CSF3 | 15941 | -1.948334575 | -0.136618 |
| PDGFB | 15994 | -2.29958725 | -0.11128437 |
| IL11 | 16032 | -2.937808275 | -0.077069804 |
| SOCS1 | 16036 | -2.972640514 | -0.04028498 |
| CSF2 | 16043 | -3.289428473 | 2.51E-04 |

Table S9.

PDX tumors.

| Tissue ID | Genetic mutations |
| --- | --- |
| PDX_LU_12 | *EGFR T790M; L747_P753>S* |
